# DFT Calculations of ^1^H- and ^13^C-NMR Chemical Shifts of Geometric Isomers of Conjugated Linoleic Acid (18:2 ω-7) and Model Compounds in Solution

**DOI:** 10.3390/molecules25163660

**Published:** 2020-08-11

**Authors:** Themistoklis Venianakis, Christina Oikonomaki, Michael G. Siskos, Panayiotis C. Varras, Alexandra Primikyri, Eleni Alexandri, Ioannis P. Gerothanassis

**Affiliations:** Section of Organic Chemistry and Biochemistry, Department of Chemistry, University of Ioannina, GR-45110 Ioannina, Greece; vethemis@gmail.com (T.V.); xristinaoik7@hotmail.com (C.O.); panostch@gmail.com (P.C.V.); aleprimik@gmail.com (A.P.); alexandri_e@hotmail.com (E.A.); igeroth@uoi.gr (I.P.G.)

**Keywords:** CLA, chemical shifts, DFT, GIAO, NMR

## Abstract

A density functional theory (DFT) study of the ^1^H- and ^13^C-NMR chemical shifts of the geometric isomers of 18:2 ω-7 conjugated linoleic acid (CLA) and nine model compounds is presented, using five functionals and two basis sets. The results are compared with available experimental data from solution high resolution nuclear magnetic resonance (NMR). The experimental ^1^H chemical shifts exhibit highly diagnostic resonances due to the olefinic protons of the conjugated double bonds. The “inside” olefinic protons of the conjugated double bonds are deshielded than those of the “outside” protons. Furthermore, in the *cis/trans* isomers, the signals of the *cis* bonds are more deshielded than those of the *trans* bonds. These regularities of the experimental ^1^H chemical shifts of the olefinic protons of the conjugated double bonds are reproduced very accurately for the lowest energy DFT optimized single conformer, for all functionals and basis sets used. The other low energy conformers have negligible effects on the computational ^1^H-NMR chemical shifts. We conclude that proton NMR chemical shifts are more discriminating than carbon, and DFT calculations can provide a valuable tool for (i) the accurate prediction of ^1^H-NMR chemical shifts even with less demanding functionals and basis sets; (ii) the unequivocal identification of geometric isomerism of CLAs that occur in nature, and (iii) to derive high resolution structures in solution.

## 1. Introduction

High resolution nuclear magnetic resonance (NMR) spectroscopy has become a widely utilized method in structure elucidation and in the qualitative and quantitative analysis of lipid molecules [1,2,3,4,5,6]. NMR spectroscopy, even in mixture analysis, has several advantages compared to common chromatographic analysis methods, since it is non-destructive, no specific standards are necessary for quantification, and chemical shifts and coupling constants are a function of the nucleus and its environment. NMR-based analytical and structural methods in lipid research, however, are limited by overlapping signals in the ^1^H-NMR spectrum, the low natural abundance of ^13^C, and the high cost of instrumentation. Nevertheless, high-resolution NMR spectroscopy has become increasingly popular in the study of lipids due to immense advances in high field instrumentation, the use of high sensitivity cryogenic probes, advanced data processing, improved automated methods for assignment, and the great variety of 1D and 2D NMR techniques [1,2,3,4,5,6,7,8,9,10].

**Figure 1:**
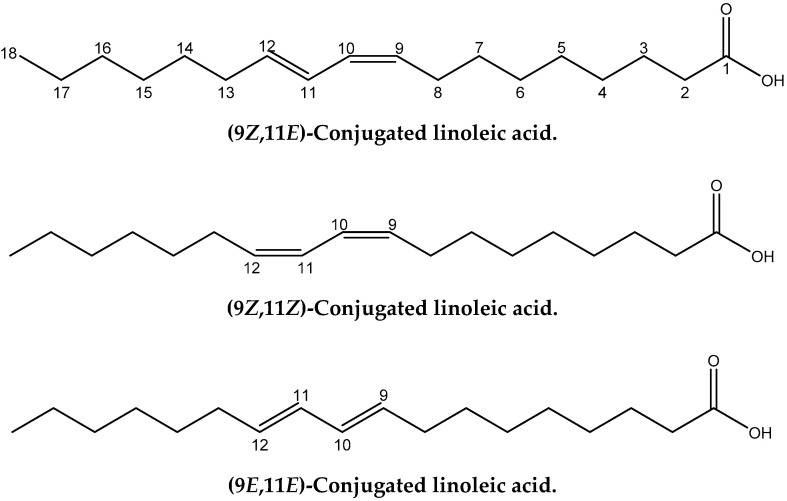

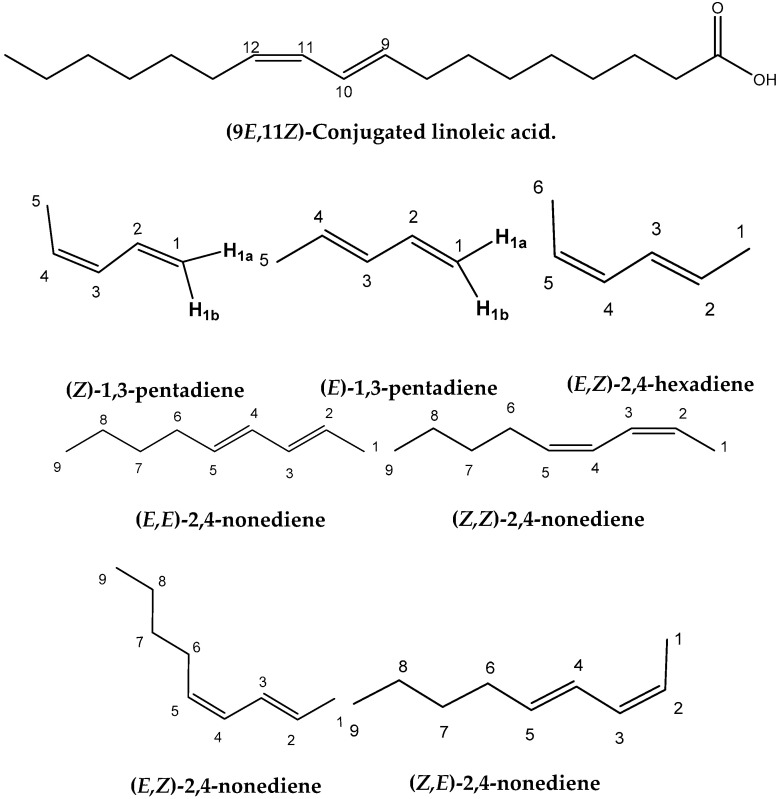
Chemical structures of the four geometric isomers of the 18:2 ω-7 conjugated linoleic acid (rumenic acid) and the model compounds investigated in the present work.

Conjugated linoleic acids (CLAs) refer to an important class of polyunsaturated fatty acids that are a mixture of geometric (*cis-cis, trans-trans, cis-trans and trans-cis*) and positional (6–8 to 13–15) fatty acid isomers that contain conjugated double bonds [11]. CLA isomers occur naturally in foods derived from ruminants and are produced as intermediates of the biohydrogenation of polyunsaturated fatty acids, especially linoleic (*cis*-9, *cis*-12) 18:2 and α-linolenic (*cis*-9, *cis*-12, *c*is-15) 18:3 by rumen bacteria [12]. *Cis*-9, *trans*-11 CLA (Figure 1) is also called rumenic acid and it accounts for 72–94% of the total CLA in foods from ruminant animals. CLAs have numerous effects with regard to cancer, atherosclerosis, obesity, and immune response [13]. Thus, it has been claimed that the *cis*-9, *trans*-11 18:2 CLA isomer, in a range of animal models, inhibits chemically induced cancer [14] and cancer cell proliferation [15], and is implicated in immunomodulation and anti-atherosclerosis effects. On the contrary, there is evidence that the *trans*-10, *cis*-12 18:2 CLA isomer may adversely affect insulin sensitivity and result in pro-carcinogenic effects in animal models [16,17].

Conjugated linoleic acids (CLAs) have been extensively investigated using ^1^H- and ^13^C-NMR [6,18,19,20,21,22]. NOESY experiments, and the analyses of spin–spin coupling constants were utilized to identify the geometric configurations of the double bonds [23]. The DFT calculations of NMR chemical shifts in conjugated systems, however, are limited to ^1^H-NMR chemical shifts and ^13^C chemical shift tensors of all-*trans*, 13-*cis*, 11-*cis*-12-*s-c*is, 11-*cis*-12-*s-trans,* and 9-*cis* isomers of retinal using the gauge including atomic orbital (GIAO) technique in combination with the B3LYP/6-31G method [24]. It was concluded that the DFT theory provides a valuable tool for the prediction of ^13^C- NMR properties, even with a small basis set. For the accurate prediction of trends in the experimental ^1^H-NMR chemical shifts a larger basis set, including polarization functions, was recommended [24].

A significant number of studies have been published that combine experimental NMR chemical shifts with computations [24,25,26,27,28] and investigating high resolution structures in solution [28,29,30,31,32,33,34,35,36]. When we consider the importance of calculations of NMR parameters in structural chemistry, it is surprising that no applications in lipid research have so far been reported. Furthermore, since no X-ray structures of CLAs have so far been published, it would be of interest to utilize the quantum chemical calculations of the chemical shifts of structure elucidation in solution of this important class of natural products. In this paper we discuss: (i) the accuracy of the calculation of ^1^H- and ^13^C-NMR chemical shifts performed within the DFT framework using the GIAO [37] technique on several geometric isomers of CLA and model compounds (Figure 1), and (ii) the use of ^1^H- and ^13^C- NMR chemical shifts for the unequivocal assignment of geometric isomerism of CLAs and as a tool for investigating high resolution structures in solution.

## 2. Results and Discussion

### 2.1. DFT-Calculated vs. Experimental ^1^H-and ^13^C-NMR Chemical Shifts of Model Compounds in Solution: Effects of Various Functionals and Basis Sets

#### 2.1.1. ^1^H-NMR

**Figure 2:**
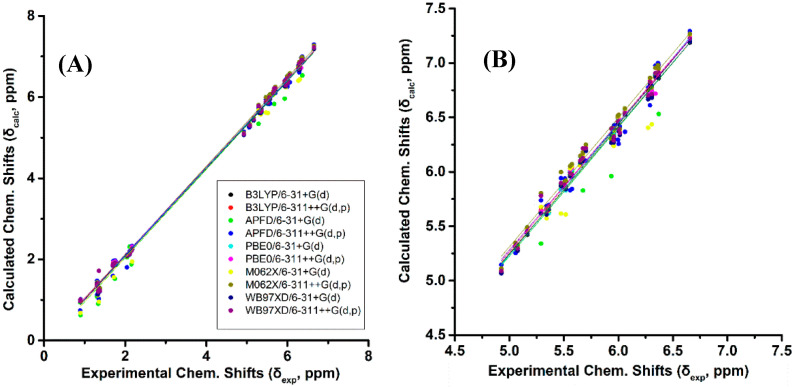
(**A**) Calculated, δ_calc_, ^1^H-NMR chemical shifts (at the GIAO/B3LYP/6-311+G(2d,p) level of theory with CPCM in CHCl_3_/CH_3_CN) vs. experimental, δ_exp_, chemical shifts with energy minimization using various functionals and basis sets for (*Z*)-1,3-pentadiene, (*E*)-1,3-pentadiene, (*E*,*Z*)-2.4-hexadiene, (*E*,*E*)-2,4-nonediene, (*Z*,*Z*)-2,4-nonediene, (*E*,*Z*)- 2,4-nonediene, and (*Z*,*E*)-2,4-nonediene (Figure 1). (**Β**) Calculated, δ_calc_, ^1^H-NMR chemical shifts of the olefinic protons vs. experimental, δ_exp_, chemical shifts of the data of Figure 2A.

Figure 2A and Table S1 show calculated, δ_calc_, (at the GIAO/B3LYP/6-311+G(2d,p) level with CPCM in CHCl_3_/CH_3_CN) vs. experimental, δ_exp_, ^1^H-NMR chemical shifts of (*Z*)-1,3-pentadiene, (*E*)-1,3-pentadiene, and (*E*,*Z*)-2.4-hexadiene in CDCl_3_ [38], and (*E,E*)-2,4-nonediene, (*Z*,*Z*)-2,4-nonediene, (*E*,*Z*)-2,4-nonediene, and (*Z*,*E*)-2,4-nonediene in CD_3_CN [39]. The optimization of the structures was performed at the B3LYP/6-31+G(d), B3LYP/6-311++G(d,p), APFD/6-31+G(d), APFD/6-311++G(d,p), PBE0/6-31+G(d), PBE0/6-311++G(d,p), M06-2X/6-31+G(d), M06-2X/6-311++G(d,p), ωB97XD/6-31+G(d), and *ω*B97XD/6-311++G(d,p) level. Excellent linear regression correlation coefficients and standard deviations of δ(^1^H) were obtained for all functionals and basis sets used, including the B3LYP/6-31+G(d) level (Table S2). This is in agreement with the conclusion of a comprehensive review article that increasing basis set and computational time does not necessarily result in more accurate chemical shifts [26]. In general, slopes that deviate from the ideal value of +1.00 by ≤0.05 and R^2^ values > 0.995 are indicative of very well performing method. Figure 2B illustrates δ_calc_ vs. δ_exp_^1^H-NMR chemical shifts of the olefinic region of Figure 2A Again, excellent linear regression correlation coefficients (≥ 0.968) were obtained with the exception of the calculations at the APFD/6-31+G(d) (R^2^ = 0.941) and M06-2X/6-31+G(d) (R^2^ = 0.938) levels (Table S2).

The experimental ^1^H-NMR chemical shifts of the model compounds of Figure 1 exhibit highly diagnostic resonances due to the –CH= protons of the conjugated double bonds in the region of 4.93 to 6.66 ppm. Some of these resonances are clearly shifted to higher frequencies than those of the isolated double bonds, thus illustrating the increased delocalization of conjugated double bonds. The “inside” olefinic protons of the conjugated double bonds are more deshielded than those of the “outside” protons. Thus, the experimental C(4)–H and C(3)–H protons of (*E*,*E*)-2,4-nonediene both appear at 6.00 ppm, while those of C(5)–H and C(2)–H appear at 5.56 and 5.57 ppm, respectively. Furthermore, in the *cis/trans* isomers the signals of the *cis* bonds are more deshielded than those of the *trans* bond. Thus, C(3)–H and C(2)–H of (*Z,E*)-2,4-nonediene appear at 5.96 and 5.36 ppm, respectively, while those of C(4)–H and C(5)–H appear at 6.36 and 5.68 ppm, respectively. In the case of identical bond configuration, the two “inside” protons have very similar chemical shifts as well as the two signals of the “outside” protons.

**Figure 3:**
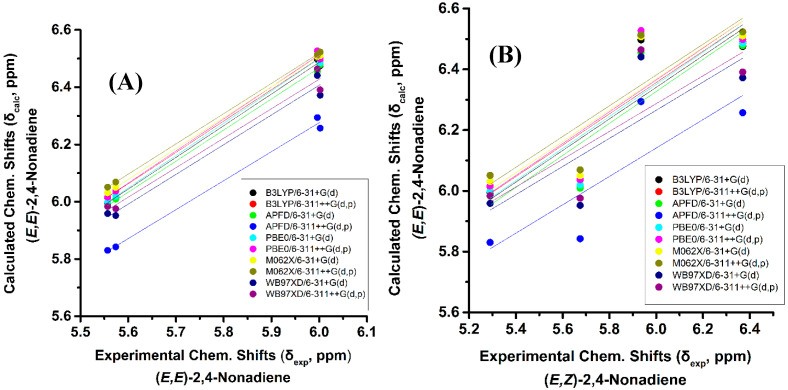
(**A**) Calculated, δ_calc_, of the olefinic protons (at the GIAO/B3LYP/6-311+G(2d,p) level of theory with CPCM in CH_3_CN) of (*E,E*)-2,4-nonediene vs. experimental, δ_exp_, olefinic protons in CD_3_CN of (*E*,*E*)-2,4-nonediene with energy minimization using the B3LYP/6-31+G(d), B3LYP/6-311++G(d,p), APFD/6-31+G(d), APFD/6-311G++(d,p), PBE0/6-31+G(d), and PBE0/6-311++G(d,p) methods. (**B**) Calculated, δ_calc_, of the olefinic protons (at the GIAO/B3LYP/6-311+G(2d,p) level of theory with CPCM in CH_3_CN) of (*E*,*E*)-2,4-nonediene vs. experimental, δ_exp_, olefinic protons in CD_3_CN of (*E*,*Z*)-2,4-nonediene with energy minimization using the same basis sets and functionals as in (**A**).

The above regularities of δ_exp_ of the olefinic protons are clearly reproduced for all functionals and basis sets used (Tables S1 and S2) and, thus can be used as criteria of the geometric isomerism of the conjugated system. Figure 3B illustrates that very poor correlation was obtained (R^2^: 0.454 to 0.657, intercept: 3.168 to 3.544, and slope: 0.457 to 0.528) when δ_calc_ of (*E*,*E*)-2,4-nonediene was plotted vs. δ_exp_ of (*E*,*Z*)-2,4-nonediene. Similarly, a very poor correlation was obtained when δ_calc_ of (*E*,*E*)-2,4-nonediene was plotted vs. δ_exp_ of (*Z*,*Z*)-2,4-nonediene (intercept: 2.626 to 2.906, and slope: 0.551 to 0.616) and when δ_calc_ of (*E*,*Z*)-2,4-nonediene was plotted vs. δ_exp_ of (*Z*,*E*)-2,4-nonediene (intercept: 3.403 to 3.922, and slope: 0.408 to 0.486) (Figures S1 and S2). It can, therefore, be concluded that ^1^H-NMR chemical shift calculations very accurately reproduce the general trends of the experimental data, and that the minor basis set dependence can result in levels of accuracy which are necessary for high resolution three-dimensional structure determinations (see below).

#### 2.1.2. ^13^C-NMR 

Figure S3 and Table S3 show calculated, δ_calc_, (at the GIAO/B3LYP/6-311+G(2d,p) level with CPCM in CHCl_3_/CH_3_CN) vs. experimental, δ_exp_, ^13^C-NMR chemical shifts of (*Z*)-1,3-pentadiene, (*E*)-1,3-pentadiene, and (*E*,*Z*)-2,4-hexadiene in CDCl_3_ [38], and (*E*,*E*)-2,4-nonediene, (*Z,Z*)-2,4-nonediene, (*E*,*Z*)-2,4-nonediene, and (*Z*,*E*)-2,4-nonediene in CD_3_CN [39]. The optimization of the structures was performed at the B3LYP/6-31+G(d), B3LYP/6-311++G(d,p), APFD/6-31+G(d), APFD/6-311++G(d,p), PBE0/6-31+G(d), PBE0/6-311++G(d,p), M06-2X/6-31+G(d), M06-2X/6-311++G(d,p), ωB97XD/6-31+G(d), and ωB97XD/6-311++G(d,p) level. Excellent linear regression correlation coefficient (R^2^: 0.997 to 0.996) was obtained, however, the mean square error (7.876 to 12.742), intercept (2.674 to 4.237), and slope (1.026 to 1.032) differed markedly from the ideal values for all functionals and basis sets used (Table S4). Figure S3B illustrates δ_calc_ vs. δ_exp_^13^C-NMR chemical shifts of the olefinic region of Figure S3A. Poor linear regression correlation coefficients (R^2^: 0.873 to 0.911) were obtained for the functional and basis sets used. The mean square error (3.568 to 5.015), intercept (−13.154 to −23.743), and slope (1.160 to 1.242) of δ_calc_ vs. δ_exp_ differed markedly from the ideal values, which is a serious deficiency in any predictive scheme (Table S5). 

### 2.2. Effects of Out-of-Plane Deformation of the Conjugated System and Conformation of Substituents on the Calculated ^1^H- and ^13^C-NMR Chemical Shifts in Model Compounds

#### 2.2.1. ^1^H-NMR 

**Figure 4:**
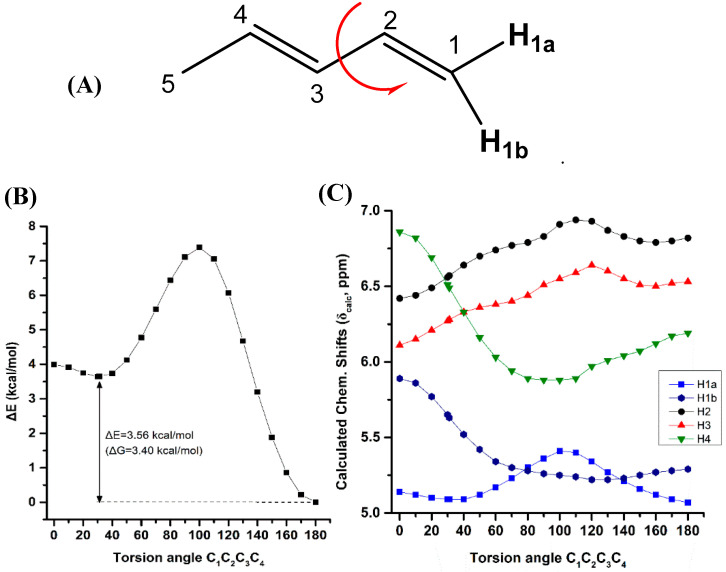
Effect of variation of the C_1_C_2_C_3_C_4_ torsion angle of (*E*)-1,3-pentadiene (**A**) on the electronic energy ΔΕ (kcal·mol^−1^) (**B**), and olefinic ^1^H-NMR chemical shifts (**C**) with energy minimization at the B3LYP/6-31+G(d) level. The Gibbs energy difference, ΔG, of the two low energy conformers is also indicated.

Variation of the torsion angle φ(C-1,C-2,C-3,C-4) of the conjugated system of (*Z*)-1,3-pentadiene in steps of 5° in the range of 180° to 0°, results in a significant increase in the electronic energy (ΔE) (Figure 4B). A second minimum at φ = 31.0° (s-*cis* conformer) was observed with ΔE = 3.56 kcal·mol^−1^ (ΔG = 3.40 kcal·mol^−1^) higher than that of the s-*trans* conformer. Table S6 shows that the computed ^1^H-NMR chemical shifts of the two low energy conformers with φ = 31.0° and φ = 180.0°, weighting by the respective Boltzmann factor, are nearly identical with those of the φ = 180.0° conformer. The effect of the population of the φ = 31.0° conformer can thus be neglected. Conjugation of C=C double bonds strengthens the anisotropy effect which can be quantitatively defined by the extent of π-electron delocalization [40]. A more pronounced π-delocalization along the conjugated double bonds results in a stronger overall anisotropy effect (*c.f.* φ = 0° vs. φ = 180°). The computational data indicate a similar behavior of the “inside” H2 and H3 protons with a pronounced shielding at φ < 90.0° (Figure 4C). The “outside” H1b and H4 protons show a similar characteristic Karplus-like variation with a strong deshielding at φ < 60.0°. Surprisingly, the effect on the olefinic ^1^H-NMR chemical shifts in the range of 150.0° < φ < 180.0° is very small and thus can be neglected. Interestingly, in this range of torsion angles the “inside” H2 and H3 protons are significantly deshielded than those of the “outside” H1b and H4 protons, in excellent agreement with the experimental data of the minimum energy conformer with φ = 180.0°.

**Figure 5:**
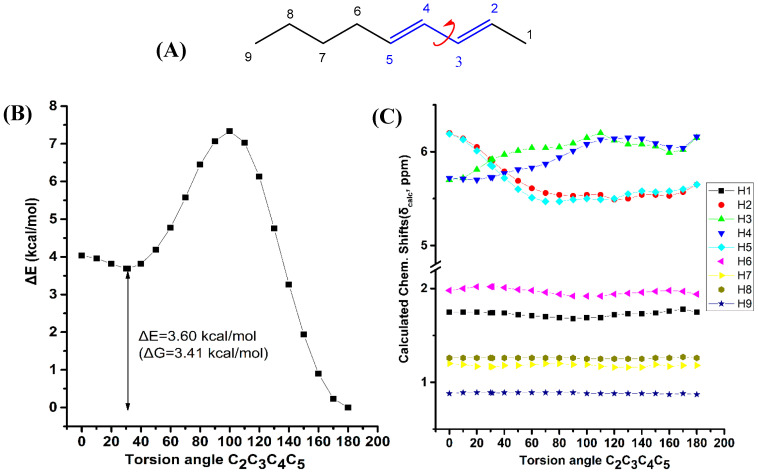
Effect of variation of the C_2_C_3_C_4_C_5_ torsion angle of *(E*,*E)*-2,4-nonediene (**A**) on the electronic energy ΔΕ(kcal·mol^−1^) (**B**), and the ^1^H-NMR chemical shifts (**C**) with energy minimization at the B3LYP/6-31+G(d) level. The Gibbs energy difference, ΔG, of the two low energy conformers is also indicated.

Figure 5 illustrates the effect of variation of the C_2_C_3_C_4_C_5_ torsion angle of (*E*,*E*)-2,4-nonediene on the electronic energy ΔΕ(kcal·mol^−1^), and olefinic ^1^H chemical shifts with energy minimization at the B3LYP/6-31+G(d) level. As in the case of (*Z*)-1,3-pentadiene, a second minimum at φ = 31.2° was observed (s-*cis* conformer) with ΔE = 3.60 kcal·mol^−1^ (ΔG = 3.41 kcal·mol^−1^) higher than that of the s-*trans* conformer (φ = 180.0°). Table S7 shows that the computed ^1^H-NMR chemical shifts of the two low energy conformers with φ = 31.2° and φ = 180.0°, weighting by the respective Boltzmann factors, are essentially the same with those of the φ = 180.0° conformer. This is due to the negligible population of the higher minimum conformer with φ = 31.2°. The “inside” olefinic protons are significantly more deshielded with respect to those of the “outside” protons for a wide range of torsion angles 60° < φ < 180° (Figure 5C). For <40° a significant cross-over of the chemical shifts is observed which results in the deshielding of the “outside” protons with respect to the “inside” protons, contrary to the experimental data (Table S1).

The shielding changes due to variation of the C_1_C_2_C_3_C_4_ torsion angle of (*Z*)-1,3-pentadiene (Figure 4) are significantly larger than those of the C_2_C_3_C_4_C_5_ torsion angle of (*E*,*E*)-2,4-nonediene. It can be concluded that although the shielding tendencies remain the same for both compounds, the C(1)-CH_3_ substitution of (*E,E*)-2,4-nonediene results in a significant reduction in the shielding ranges.

**Figure 6:**
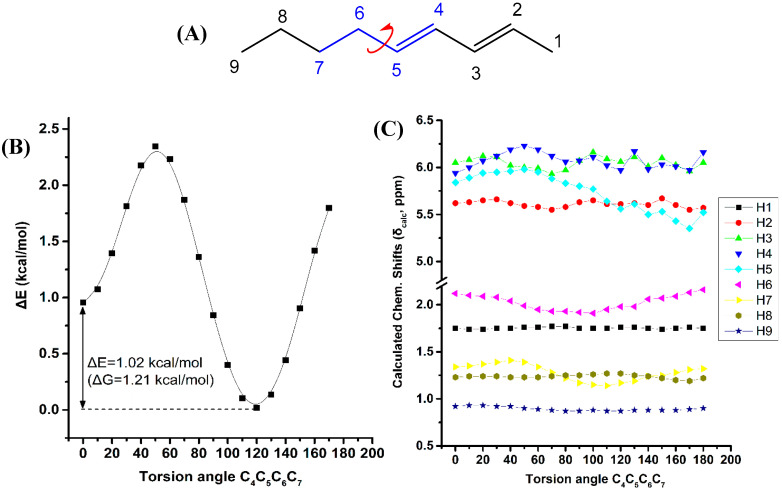
Effect of variation of the C_4_C_5_C_6_C_7_ torsion angle of *(E*,*E)*-2,4-nonediene (**A**) on the electronic energy ΔΕ(kcal·mol^−1^) (**B**), and the ^1^H-NMR chemical shifts (**C**) with energy minimization at the B3LYP/6-31+G(d) level. The Gibbs energy difference, ΔG, of the two low energy conformers is also indicated.

Figure 6 illustrates the effect of variation of the C_4_C_5_C_6_C_7_ torsion angle of the (*E*,*E*)-2,4-nonediene on the electronic energy ΔΕ, and the ^1^H-NMR chemical shifts with energy minimization at the B3LYP/6-31+G(d) level. Two low energy conformers with φ = 120.0° and φ = 0.1° were observed with ΔE = 1.02 kcal·mol^−1^ (ΔG = 1.21 kcal·mol^−1^). For a wide range of torsion angles 100° < φ < 180°, the “inside” protons are more deshielded than the “outside” protons. The experimental ^1^H-NMR chemical shifts of the olefinic protons (Table S1) are in excellent agreement with the calculated chemical shifts of the minimum energy conformer with φ = 120.0°. For φ = 0.1°, the chemical shift of the “outside” H5 becomes similar to that of the “inside” H4 proton, contrary to the experimental data. Table S7 shows that the computed ^1^H-NMR chemical shifts of the low energy conformers, weighting by the respective Boltzmann factor, are essentially the same with those of the φ = 120.0° conformer. In this case, the weights of the higher minimum conformer are not vanishing, but the respective chemical shifts of the φ = 120.0° and φ = 0.1° conformers are not significantly different. A systematic investigation of ^1^H chemical shifts in alkenes by Abraham et al. [41] showed that the ^1^H chemical shifts of olefins are influenced by both diamagnetic anisotropy and steric effects of the double bonds. There is a deshielding above the C=C bond at small distances due to the van der Waals term and shielding at large distances due to the bond anisotropy. The plane of the C=C bond there is always a deshielding effect [42]. The olefinic group has significant γ effects on protons three bonds away. This is in agreement with our results of the significant effect of the C_4_C_5_C_6_C_7_ torsion angle on the computed ^1^H-NMR chemical shifts of the H7.

**Figure 7:**
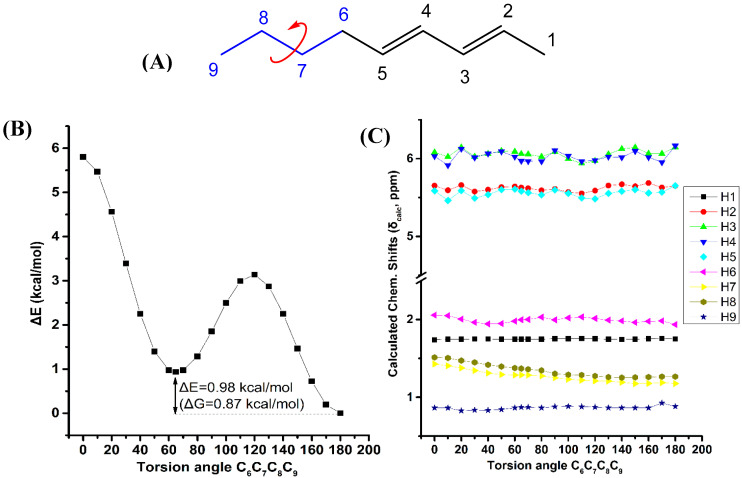
Effect of variation of the C_6_C_7_C_8_C_9_ torsion angle of *(E*,*E)*-2,4-nonediene (**A**) on the electronic energy ΔΕ(kcal·mol^−1^) (**B**), and the ^1^H-NMR chemical shifts (**C**) with energy minimization at the B3LYP/6-31+G(d) level. The Gibbs energy difference, ΔG, of the two low energy conformers is also indicated.

Figure 7 illustrates the effect of variation of the C_6_C_7_C_8_C_9_ torsion angle of the (*E_,_E*)-2,4-nonediene on the electronic energy, ΔΕ, and the ^1^H-NMR chemical shifts with energy minimization at the B3LYP/6-31+G(d) level. Two minima in the electronic energy were observed at 180.0° and 64.8°, with ΔE = 0.98 kcal·mol^−1^ (ΔG = 0.87 kcal·mol^−1^). In this case the weights of the higher minimum conformer are not vanishing, however, the H7 and H8 chemical shifts exhibit a parallel pattern as a function of the C_6_C_7_C_8_C_9_ torsion angle with very small chemical shift difference. This indicates that δ(H7) and δ(H8) are of minor importance in conformational analysis. 

From the above it can be concluded that the experimental ^1^H-NMR chemical shifts are reproduced very accurately for the lowest energy DFT optimized single conformer. The other low energy conformers have negligible effects on the computational ^1^H-NMR chemical shifts.

**Figure 8:**
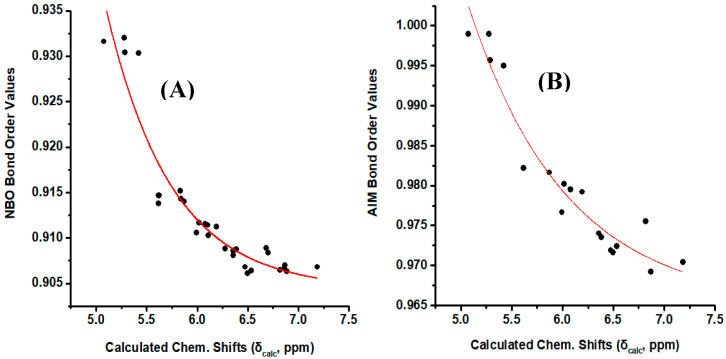
(**A**) NBO bond order of the olefinic C–H bonds of the model compounds of Figure 1 vs. calculated, δ_calc_, ^1^H-NMR chemical shifts with energy minimization at the B3LYP/6-31+G(d) level. (**B**) AIM bond order of the olefinic C–H bonds of the model compounds (*Z*)-1,3-pentadiene, (*E*)-1,3 pentadiene, (*E,Z*)-2,4 hexadiene, (*E,E*)-2,4 nonadiene vs. calculated, δ_calc_, ^1^H-NMR chemical shifts with energy minimization at the B3LYP/6-31+G(d) level.

Figure 8A illustrates a plot of NBO bond order of the olefinic C–H bonds vs. δ_calc_(^1^H). The resulting good correlation (R^2^ = 0.903) demonstrate that the NBO bond order is a primary factor which determines δ_calc_(^1^H). Similar correlation was obtained when the AIM bond order [43] was plotted vs. δ_calc_(^1^H) (Figure 8B). On the contrary, no functional relationship was found for both NBO and AIM charge densities of the olefinic C-H protons vs. δ_calc_(^1^H).

#### 2.2.2. ^13^C-NMR

Figure S4 illustrates the effect of the out-of-plane deformation of the conjugated system on the ^13^C chemical shifts of (*E*)-1,3-pentadiene and *(E*,*E)*-2,4-nonediene, respectively, with calculations at the B3LYP/6-31+G(d) level. For the *(E*,*E)*-2,4-nonediene, the “inside” carbons C-3 and C-4 indicate a similar dependence with a rather complex cross-over with C-2. Figure S5 illustrates a very significant effect of variation of the C_4_C_5_C_6_C_7_ torsion angle of *(E*,*E)*-2,4-nonediene on the ^13^C chemical shifts of the olefinic C-4 and the aliphatic C6 and C7 carbons. However, the low energy conformers with φ = 120.0° and 0.1° are characterized with minimum chemical shifts differences. From the above it can be concluded that ^13^C-NMR chemical shifts are less discriminating than ^1^H, also considering the statistical data of Tables S2, S4 and S5. Similar conclusions can be drown from the effect of variation of the C_6_C_7_C_8_C_9_ torsion angle of *(E*,*E)*-2,4-nonediene on the ^13^C chemical shifts (Figure S5).

### 2.3. DFT-Calculated vs. Experimental ^1^H-NMR Chemical Shifts: 3D-Structures of Geometric Isomers of 18:2 ω-7 CLA in Solution

The ^1^H-NMR spectra of the four geometric isomers of 18:2 ω-7 CLA exhibit highly diagnostic resonances due to the –CH= protons of the conjugated double bonds in the region of 6.34 to 5.27 ppm. As in the case of the model compounds of Figure 1, the “inside” olefinic protons of C10 and C11 are more deshielded than those of the olefinic protons of C9 and C12. Furthermore, in the *cis/trans* isomers the signals of the *cis* bonds are more deshielded than those of the *trans* bonds. In the case of identical bond configuration, the two “inside” protons have very similar chemical shifts as well as the two signals of the “outside” protons (Table S8). Figure S6 illustrates the olefinic region of the ^1^H-NMR spectra of (9*Z*,11*E*)*-*CLA in CDCl_3_ (ε = 4.81), CD_3_CN (ε = 38.8) and DMSO-d_6_ (ε = 46.7) solution. Despite the use of three solvents with significantly different dielectric constants, ε, solvation and hydrogen bonding ability, the olefinic proton chemicals shifts are essentially solvent independent. The carboxylic proton was found to be extremely broad due to the fast proton exchange rate with traces of H_2_O in the organic solvents. The resulting chemical shifts in DMSO-d_6_ (δ = 11.96), CD_3_CN (δ = 8.82), and CDCl_3_ (δ = 10.50) (Table S9) clearly demonstrate that hydrogen bond between the COOH group and the DMSO-d_6_ molecule is more efficient than in CD_3_CN. In CDCl_3_, a strong deshielding is observed due to the presence of dimeric hydrogen bonded species. However, the chemical shift is smaller than that of the dimeric acetic acid in CDCl_3_ (δ = 11.51 ppm, concentration 160 mM [44]), due to the equilibrium between dimeric and monomeric species at the concentration used (25 mM). 

Table 1 shows ΔG values (kcal·mol^−1^) and % populations of the various low energy conformers of the geometric isomers of 18:2 ω-7 CLA in the gas phase with energy minimization at the B3LYP/6-31+G(d) and APFD/6-31+G(d) level. According to the most stable conformation of the butyl group in 2,4-nonediene geometric model isomers, a similar conformational behavior is expected for the two alkyl groups which are attached to the conjugated system of linoleic acid. Consequently, a variety of five low energy conformers was obtained. In Figures S7–S11 the structures of five conformers (A), (B), (C), (D), and (E) of the four geometric isomers of 18:2 ω-7 CLA, are presented.

Figure S12 shows calculated (at the GIAO/WP04/6-311+G(2d,p) (CPCM, CHCl_3_) level of theory) vs. experimental ^1^H-NMR chemical shifts of the four 9,11-conjugated linoleic acid geometric isomers with optimization of the structures at the B3LYP/6-31+G(d), and APFD/6-31+G(d) level of theory. The resulting statistical data of Table S10 demonstrate that computational ^1^H-NMR chemical shifts reproduce accurately the general trends of the experimental data. Very good correlations were also obtained when a discrete solvation molecule was utilized to model hydrogen bond interaction of the COOH group of the (9*Z*,11*E*)-CLA in DMSO and CH_3_CN solution (Figure S13). The dimeric form in CDCl_3_ was modeled with (9*Z*,11*E*)-CLA complexed with a molecule of CH_3_COOH to facilitate computations. In all cases, solvation of the COOH group induces minor changes in conformation (Figures S8 and S14), in excellent agreement with the experimental ^1^H-NMR chemical shift data of Table S9.

A typical workflow that can be used for investigating homologous CLA compounds includes the following steps:

(i) The ^1^H-NMR spectra are recorded in CDCl_3_ solution at 298 K, and the experimental chemical shifts, δ_exp_, are determined.
(ii) The ^1^H-NMR chemical shifts are computed at the GIAO B3LYP/6-311+G(2d,p) level with the CPCM model with energy minimization using the B3LYP/6-311++G(d,p) method.
(iii) A very good linear correlation between experimental NMR chemical shifts, δ_exp_, and calculated δ_calc_ provides strong indication that the computational procedure is working.

## 3. Materials and Method

### 3.1. Chemicals

Conjugated (9*Z*,11*E*) 18:2 linoleic acid, purity ≥96% (HPLC) was purchased from Fluka Chemie GmbH (Buchs, Switzerland). CDCl_3_, DMSO-d_6_, and CD_3_CN were purchased from Deutero (Kastellaun, Germany).

### 3.2. NMR

The NMR experiments were performed on a Bruker AV-500 MHz, Bruker (Billerica, MA, USA). Samples of (9*Z*,11*E*)-CLA were dissolved in 0.6 mL of deuterated solvent (25 mM) and transferred to 5 mm NMR tubes. Chemical shifts were measured with reference to the residual ^1^H signal of the incompletely deuterated solvent.

### 3.3. Computational Methods

The computational study was performed by using the Gaussian 09 with the DFT method [45]. The structures of the model compounds were optimized at the B3LYP/6-31+G(d,p), B3LYP/6-311++G(d,p), APFD/6-31+G(d), APFD/6-311++G(d,p), PBE0/6-31+G(d), PBE0/6-311++G(d,p), M06-2X/6-31+G(d), M06-2X/6-311++G(d,p), ωB97XD/6-31+G(d), and ωB97XD/6-311++G(d,p) level. The structures of the (9*Z*,11*E*)-CLA geometric isomers were optimized at the B3LYP/6-31+G(d) and APFD/6-31+G(d) level. The optimized geometries were verified by performing frequency calculation at the same level (zero imaginary frequencies). The computed ^1^H- and ^13^C-NMR chemical shifts, at the GIAO/B3LYP/6-311+G(2d,p) level, of the compounds investigated, were referenced with respect to the standard TMS which was optimized at the same level. The scanning of torsional angles was performed by using the redundant coordinates in Gaussian 09.

## 4. Conclusions

From the calculated data reported herein, it can be concluded that:

(i) Excellent linear correlations can be obtained between DFT-calculated and experimental ^1^H-NMR chemical for the lowest energy DFT optimized single conformer for various functionals and basis sets, especially at the B3LYP/6-311++G(d,p) level. The other low energy conformers have negligible effects on the computational ^1^H-NMR chemical shifts.
(ii) The computational ^1^H-NMR chemical shifts can provide an unequivocal assignment of the geometric isomerism in conjugated systems of biological systems such as CLAs.
(iii) The great sensitivity of ^1^H-NMR chemical shifts to geometric isomerism and conformation of substituents can provide an excellent method for obtaining high resolution structures in solution.
(iv) The typical workflow for investigating 3D structures in solution includes a few applicable steps.

The present method of DFT calculations of ^1^H-NMR chemicals shifts in parallel with the use of the computer assisted structure elucidation (CASE) program [46,47] and the development of experimental methods [6,10,48], could be of primary importance in geometric isomer identification and high resolution structural determination not only in isolated CLAs and their primary oxidation products [49], but also in mixture analysis in the emerging field of lipidomics [50,51].

## Figures and Tables

**Table 1:** ΔG values (kcal·mol^−1^) and % populations of the various conformers of 18:2 ω-7 CLA geometric isomers in the gas phase at the B3LYP/6-31+G(d) and APFD/6-31+G(d) level.

Geometric Isomer	Optimization Method	ΔG(kcal/mol) (% Population)				
		Conformer				
		A	B	C	D	E
(9*Z*,11*E*)-CLA	B3LYP/6-31+G(d)	+0.21 (20.46)	+0.22 (20.12)	0.00 (29.16)	0.00 (29.16)	+1.94 (1.10)
	APFD/6-31+G(d)	+0.14 (23.35)	+0.38 (15.57)	0.00 (29.58)	0.00 (29.58)	+1.62 (1.92)
(9*Z*,11*Z*)-CLA	B3LYP/6-31+G(d)	0.00 (36.47)	+0.14 (28.80)	+0.03 (34.67)	+4.36 (0.02)	+4.06 (0.04)
	APFD/6-31+G(d)	+0.09 (34.27)	0.00 (39.89)	+0.26 (25.72)	+3.80 (0.06)	+3.86 (0.06)
(9*E*,11*E*)-CLA	B3LYP/6-31+G(d)	0.00 (61.55)	+0.66 (20.21)	+2.17 (1.58)	+1.18 (8.40)	+1.19 (8.26)
	APFD/6-31+G(d)	+0.99 (14.60)	0.00 (77.64)	+2.54 (1.07)	+1.96 (2.84)	+1.78 (3.85)
(9*E*,11*Z*)-CLA	B3LYP/6-31+G(d)	+0.50 (14.46)	0.00 (33.63)	+0.50 (16.55)	+0.01 (33.07)	+1.60 (2.26)
	APFD/6-31+G(d)	+0.73 (9.43)	0.00 (32.33)	+0.73 (9.43)	0.00 (32.33)	+0.40 (16.46)

## Supplementary Materials

Supplementary Materials are available online.
