# Supplementary material for: DFT Calculations of 1H- and 13C-NMR Chemical Shifts of Geometric Isomers of Conjugated Linoleic Acid (18:2 ω-7) and Model Compounds in Solution
Source: Molecules. 2020 Aug 11;25(16):3660. doi: 10.3390/molecules25163660 (PMC7463970; doi:10.3390/molecules25163660)
Supplement: Supplementary file 1 [file molecules-25-03660-s001.pdf]

molecules
Supplementary Material

DFT Calculations of ^{1}H- and ^{13}C-NMR Chemical Shifts of Geometric Isomers of Conjugated Linoleic Acid (18:2 ω-7) and Model Compounds in Solution

Themistoklis Venianakis, Christina Oikonomaki, Michael G. Siskos *, Panayiotis C. Varras, Alexandra Primikyri, Eleni Alexandri and Ioannis P. Gerothanassis

Section of Organic Chemistry and Biochemistry, Department of Chemistry, University of Ioannina, GR-45110 Ioannina, Greece; vethemis@gmail.com (T.V.); xristinaoik7@hotmail.com (C.O.); panostch@gmail.com (P.C.V.); aleprimik@gmail.com (A.P.); alexandri_e@hotmail.com (E.A.);
igeroth@uoi.gr (I.P.G.)
* Correspondence: msiskos@uoi.gr

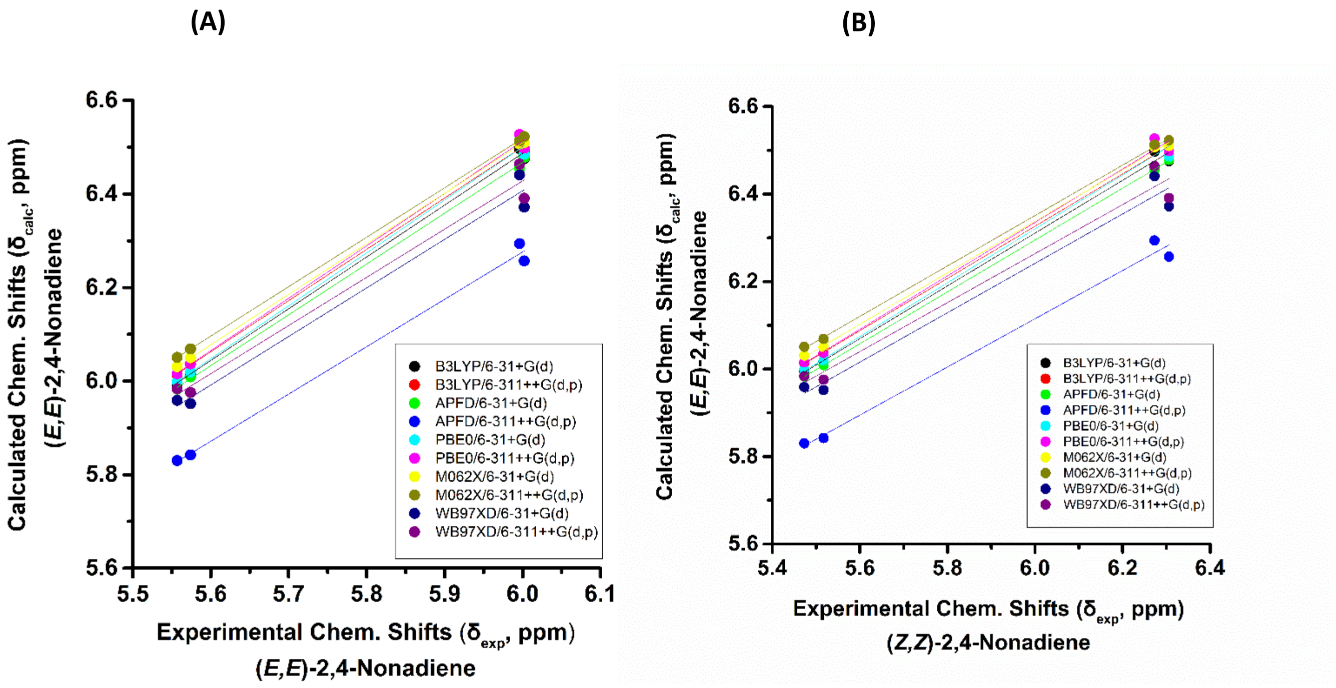

Figure 1. (A) Calculated, δ_{calc}, of the olefinic protons (at the GIAO/B3LYP/6-311+G(2d,p) level of theory with CPCM in CH_3CN) of (*E*,*E*)-2,4-nonediene *vs.* experimental, δ_{exp}, olefinic protons in CD_3CN of (*E*,*E*)-2,4-nonediene with energy minimization using the B3LYP/6-31+G(d), B3LYP/6-311++G(d,p), APFD/6-31+G(d), APFD/6-311G++(d,p), PBE0/6-31+G(d) and PBE0/6-311++G(d,p) methods. (B) Calculated, δ_{calc}, of the olefinic protons (at the GIAO/B3LYP/6-311+G(2d,p level of theory with CPCM in CH_3CN) of (*E*,*E*)-2,4-nonediene *vs.* experimental, δ_{exp}, olefinic protons in CD_3CN of (*Z*,*Z*)-2,4-nonediene with energy minimization using the same basis sets and functionals as in (A).

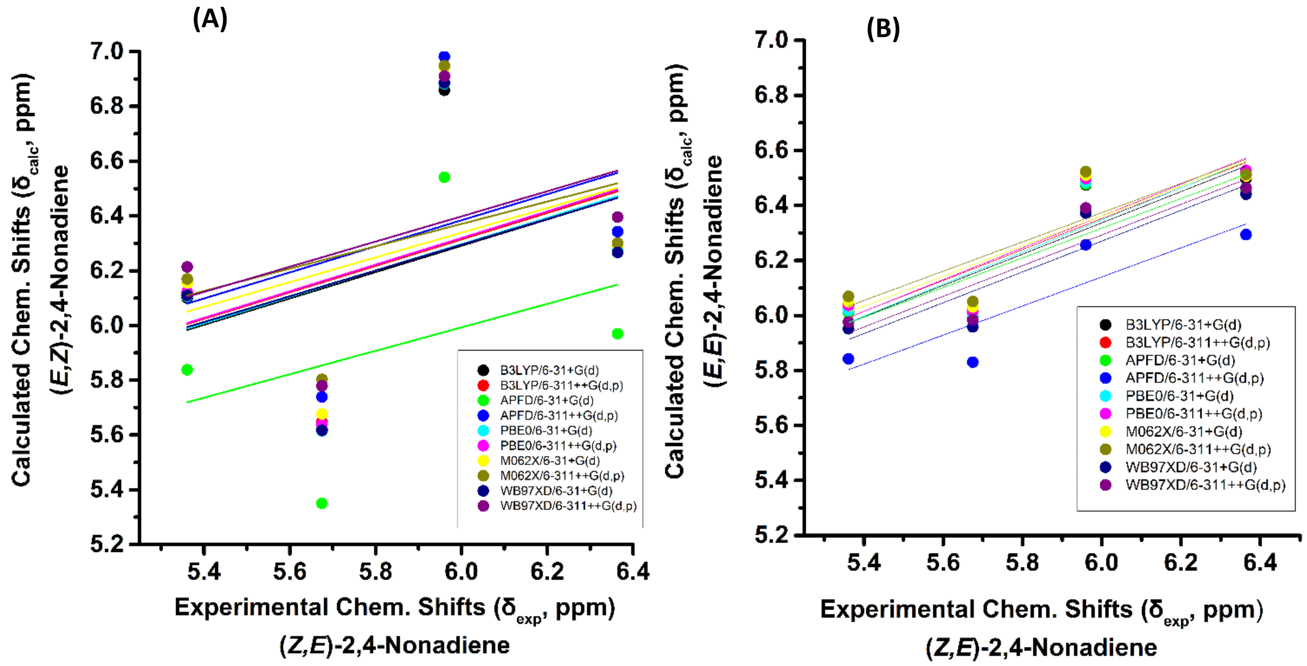

Figure 2. (A) Calculated, δ_{calc}, of the olefinic protons (at the GIAO/B3LYP/6-311+G(2d,p) level of theory with CPCM in CH_3CN) of (*E*,*Z*)-2,4-nonediene *vs.* experimental, δ_{exp}, olefinic protons in CD_3CN of (*Z*,*E*)-2,4-nonediene with energy minimization using the B3LYP/6-31+G(d), B3LYP/6-311++G(d,p),APFD/6-31+G(d), APFD/6-311G++(d,p), PBE0/6-31+G(d) and PBE0/6-311++G(d,p) methods. (B) Calculated, δ_{calc}, of the olefinic protons (at the GIAO/B3LYP/6-311+G(2d,p) level of theory with CPCM in CH_3CN) of (*E*,*E*)-2,4-nonediene *vs.* experimental, δ_{exp}, olefinic protons in CD_3CN of (*Z*,*E*)-2,4-nonediene with energy minimization using the same basis sets and functionals as in (A).

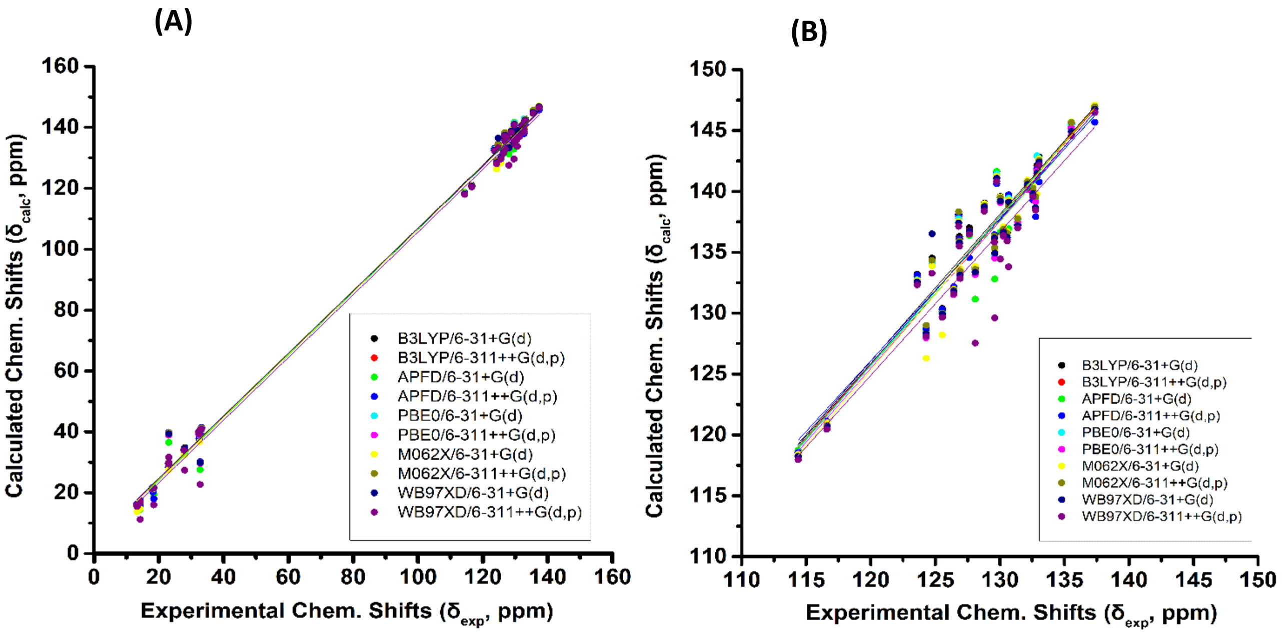

Figure 3. **(A)** Calculated, δ_{calc}, ^{13}C-NMR chemical shifts (at the GIAO/B3LYP/6-311+G(2d,p) level of theory with CPCM $CHCl_3$/CH_3CN)*vs.* experimental, δ_{exp}, chemical shifts with energy minimization using various functionals and basis sets for (*Z*)-1,3-pentadiene, (*E*)-1,3-pentadiene, (*E*,*Z*)-2,4-hexadiene, (*E*,*E*)-2,4-nonediene, (*Z*,*Z*)-2,4-nonediene, (*E*,*Z*)- 2,4-nonediene, and (*Z*,*E*)-2,4-nonediene (Fig. 1). **(B)** Calculated, δ_{calc}, ^{13}C-NMR chemical shifts of the olefinic protons (at the GIAO/B3LYP/6-311+G(2d,p) level of theory with CPCM in $CHCl_3$/CH_3CN) *vs.* experimental, δ_{exp}, chemical shifts with energy minimization using various functionals and basis sets for (*Z*)-1,3-pentadiene, (*E*)-1,3-pentadiene, (*E*,*Z*)-2.4-hexadiene, (*E*,*E*)-2,4-nonediene, (*Z*,*Z*)-2,4-nonediene, (*E*,*Z*)- 2,4-nonediene, and (*Z*,*E*)-2,4-nonediene (Fig. 1).

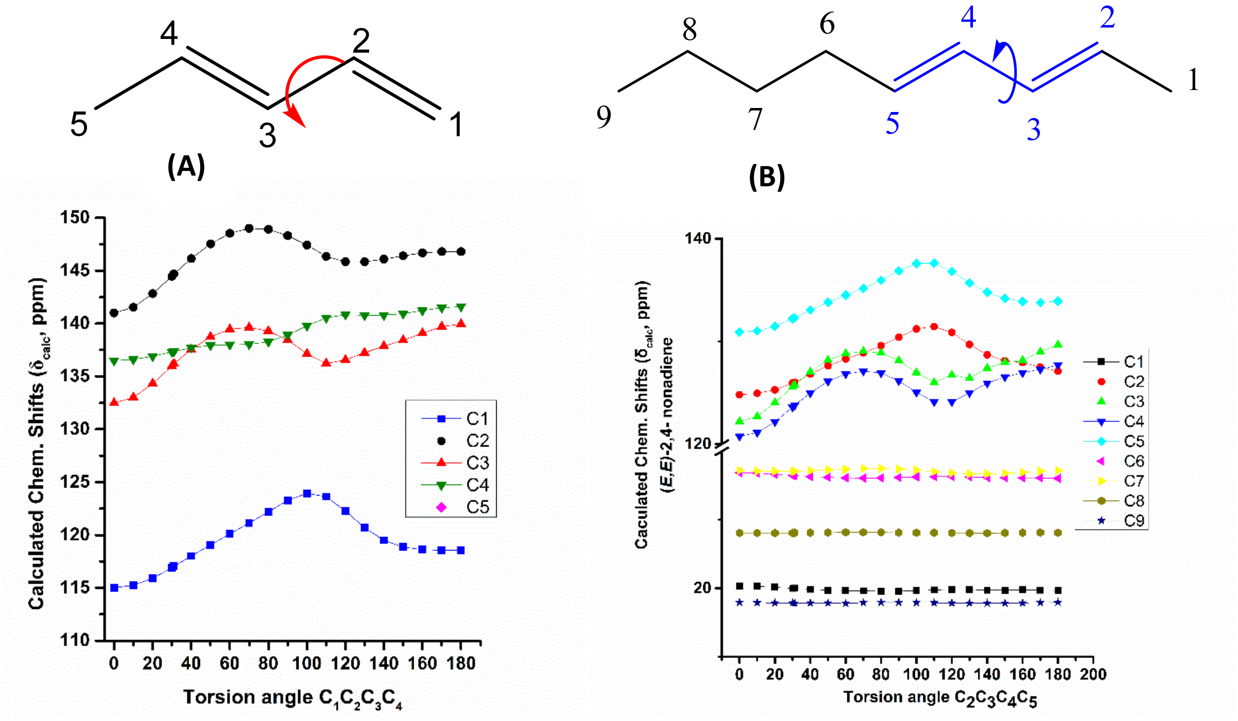

Figure 4. **(A)** Effect of variation of the $C_1C_2C_3C_4$ torsion angle on the olefinic ^{13}C chemical shifts of (*E*)-1,3-pentadiene with calculations at the B3LYP/6-31+G(d) level. **(B)** Effect of variation of the $C_2C_3C_4C_5$ torsion angle of (*E*,*E*)-2,4-nonediene on the ^{13}C chemical shifts, with energy minimization at the B3LYP/6-31+G(d) level.

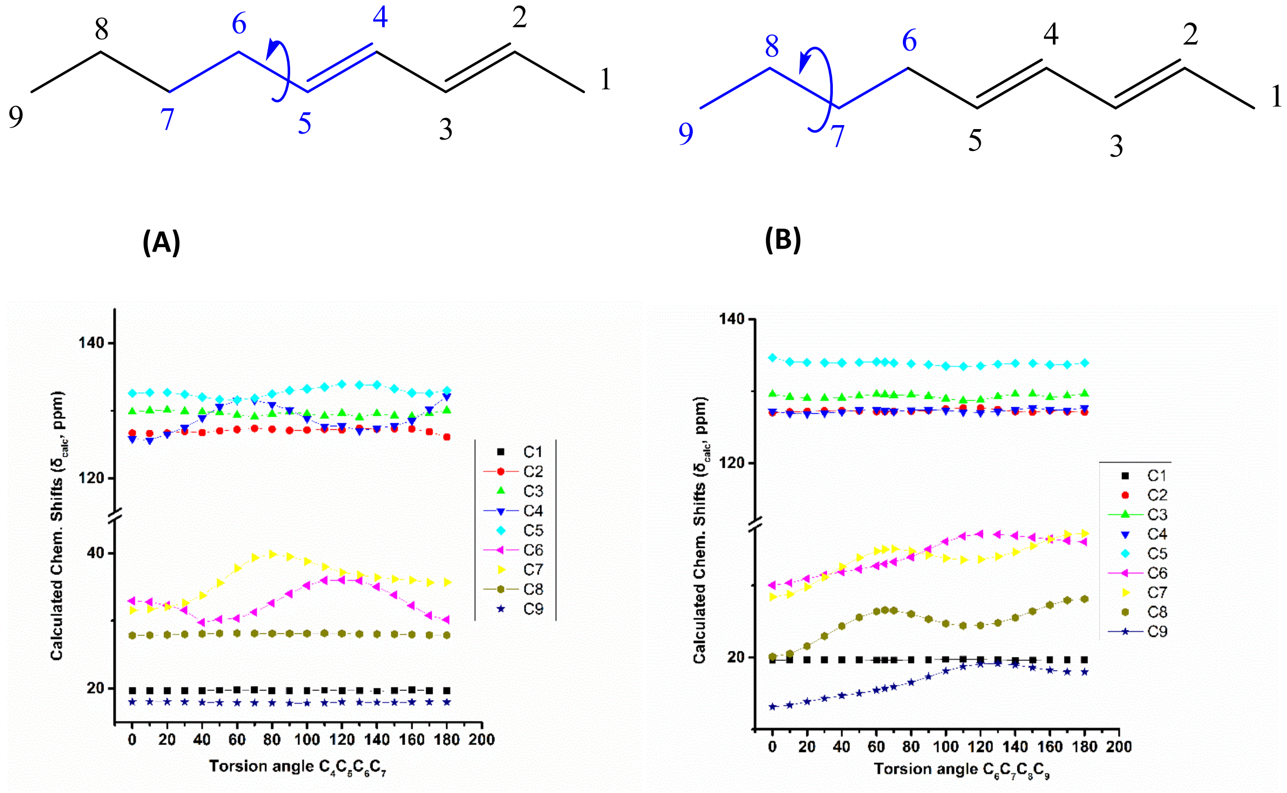

Figure 5. Effect of variation of the $C_4C_5C_6C_7$ torsion angle of (*E*,*E*)-2,4-nonediene on the ^{13}C chemical shifts, with calculations at the B3LYP/6-31+G(d) level. **(B)** Effect of variation of the $C_6C_7C_8C_9$torsion angle of (*E*,*E*)-2,4-nonediene on the ^{13}C chemical shifts, with calculations with energy minimization at the B3LYP/6-31+G(d) level.

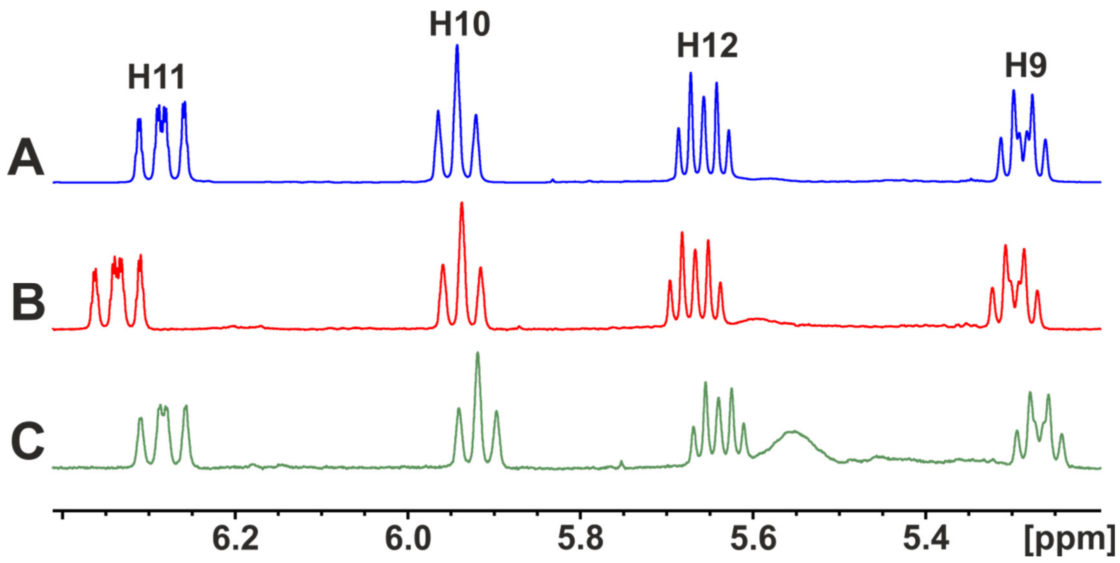

Figure 6. Selective region of the ^1H NMR spectrum of (9*Z*,11*E*)-CLA in (A) $CDCl_3$, (B) CD_3CN and (C) DMSO-d_6, 298K, number of scans = 256, acquisition time = 2.04s, relaxation delay = 5 s, total experimental time = 30 min].

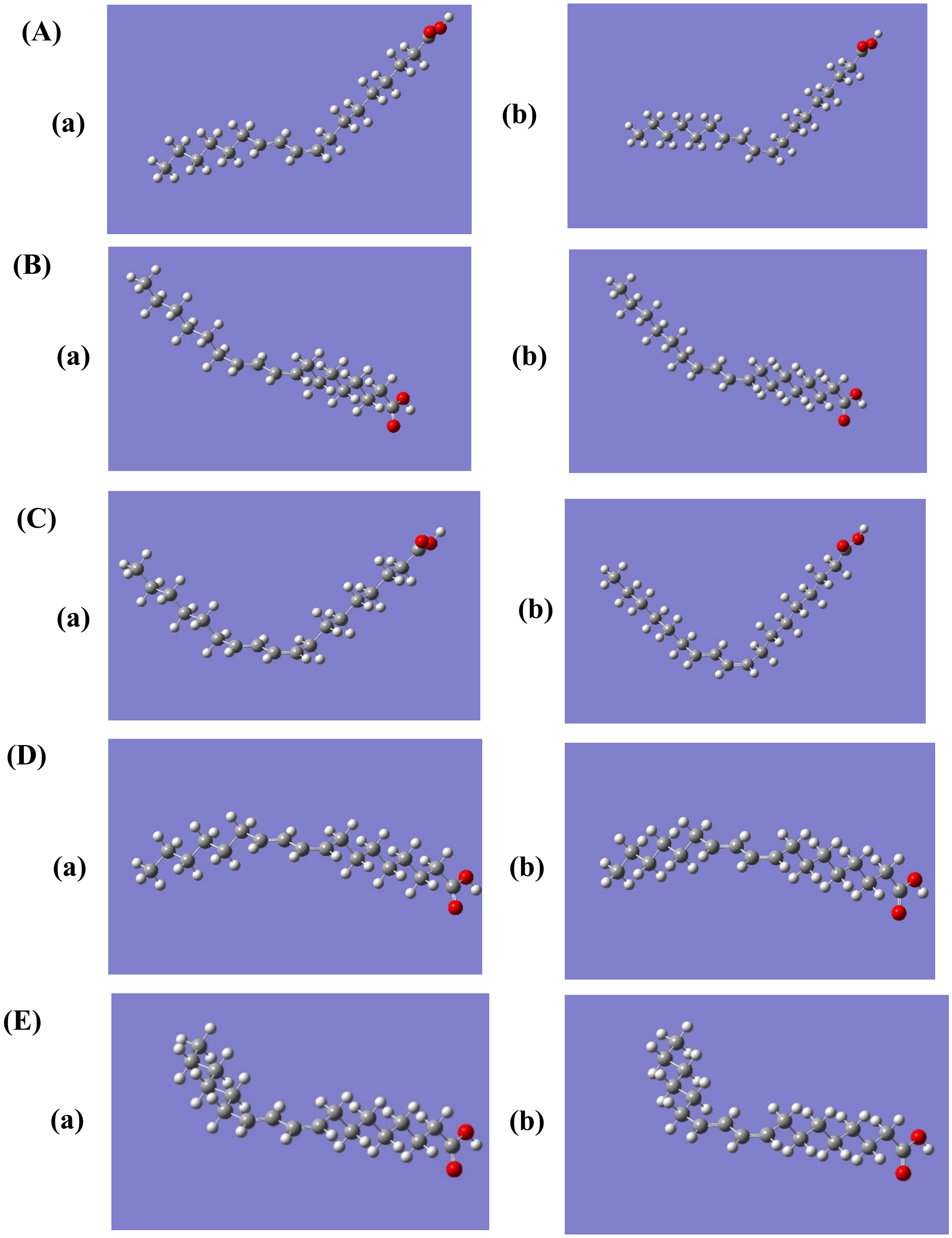

Figure 7. Structures of various conformers (A), (B), (C), (D), and (E) of the (9*Z*,11*E*)-CLA with energy minimization in the gas-phase at the B3LYP/6-31+G(d) (**a**) and APFD/6-31+G(d) (**b**) level. ΔG values (kcal.mol^{-1}) and % populations of conformers (A), (B), (C), (D), and (E) are shown in Table 1.

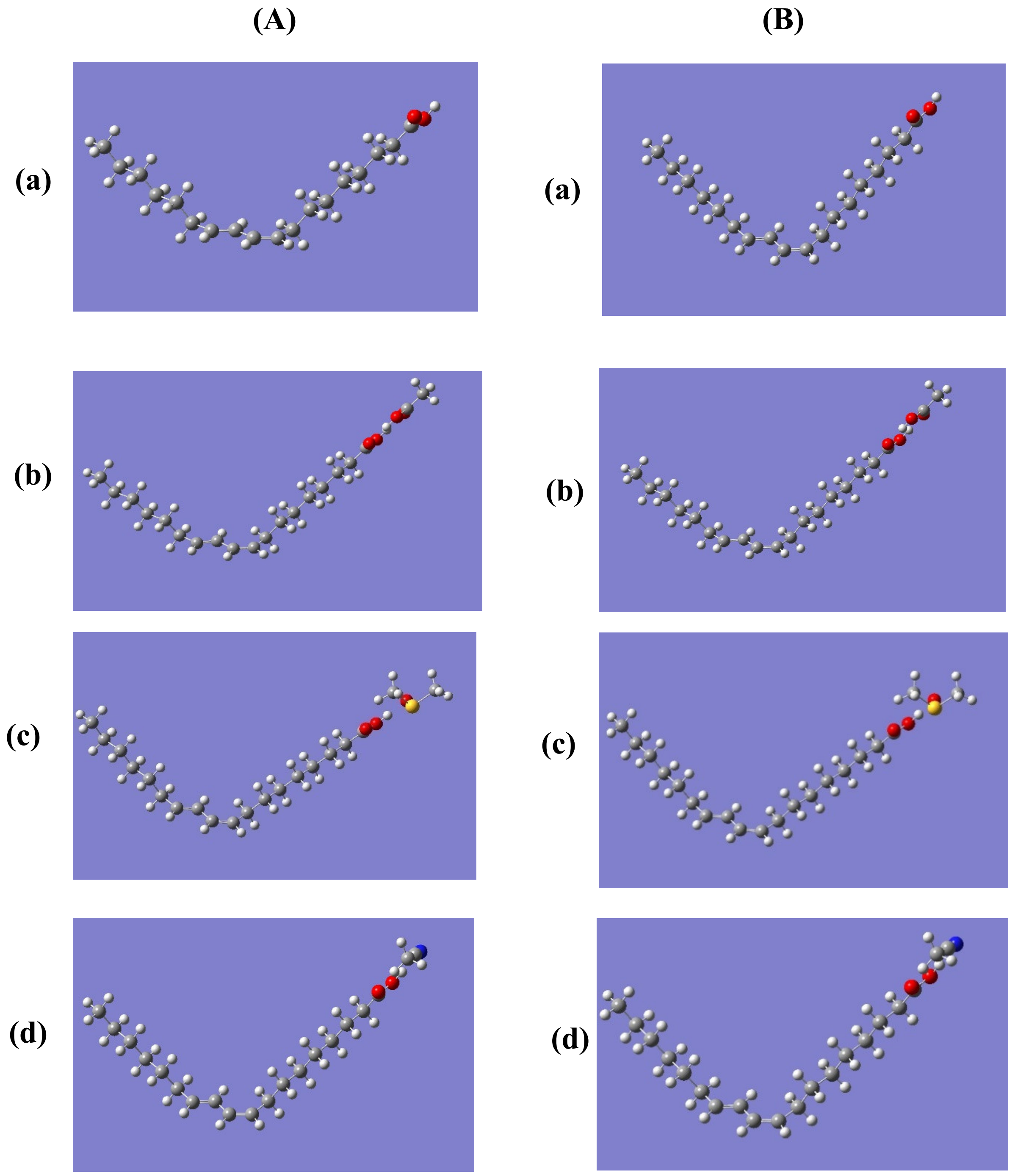

Figure 8. Effects of solvation on the structures of the low energy conformer C (Fig. S7, Table 1) of the(9*Z*,11*E*)-CLA in the gas-phase (**a**), and with a discrete molecule of CH_3COOH (**b**), DMSO (**c**) and CH_3CN (**d**), with energy minimization at the B3LYP/6-31+G(d) (A) and APFD/6-31+G(d) (**B**) level.

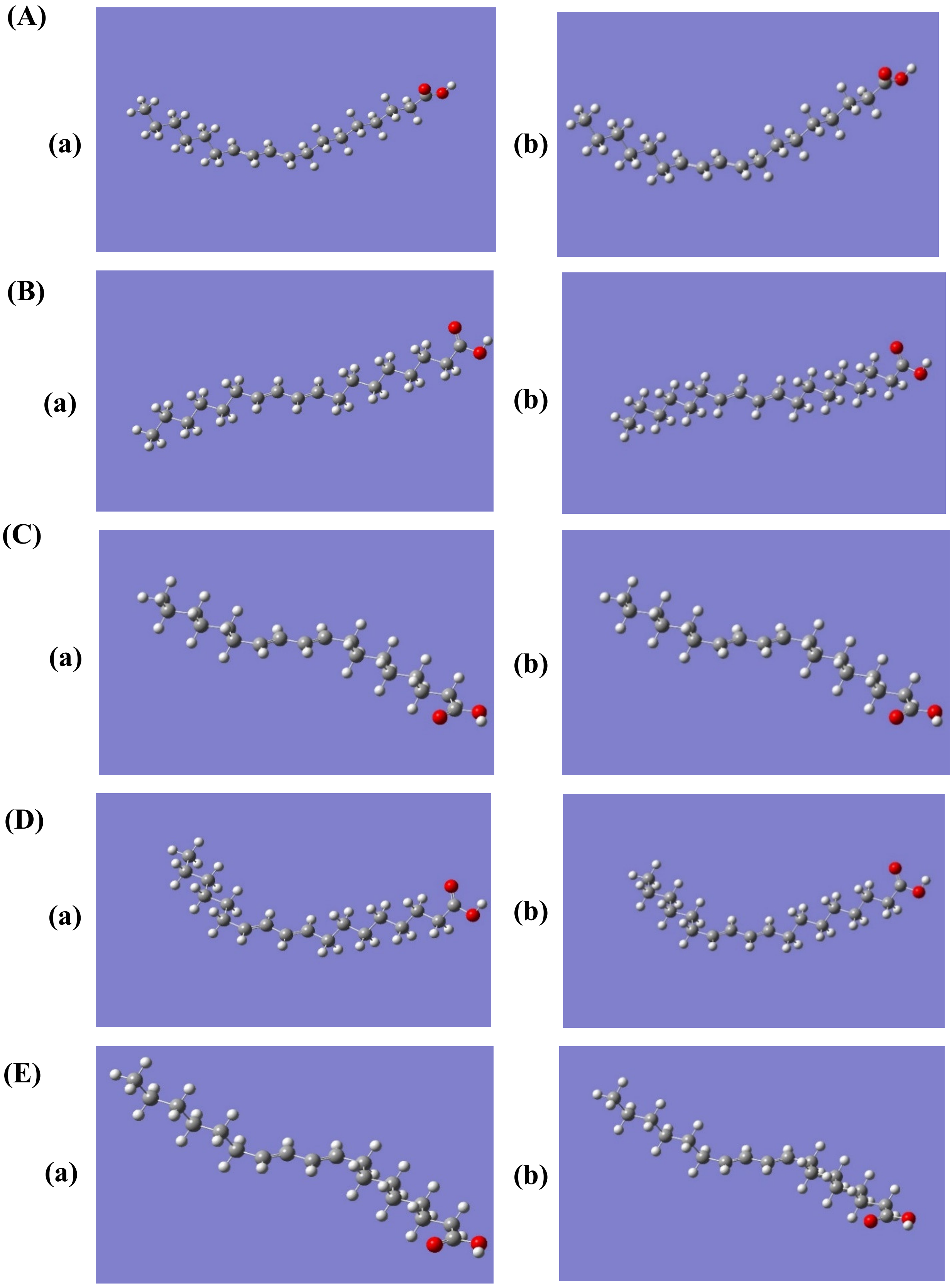

Figure S9. Structures of various conformers (A), (B), (C), (D), and (E) of the (9*E*,11*E*)-CLA with energy minimization in the gas-phase at the B3LYP/6-31+G(d) (**a**) and APFD/6-31+G(d) (**b**) level. ΔG values (kcal.mol^{-1}) and % populations of conformers (A), (B), (C), (D), and (E) are shown in Table 1.

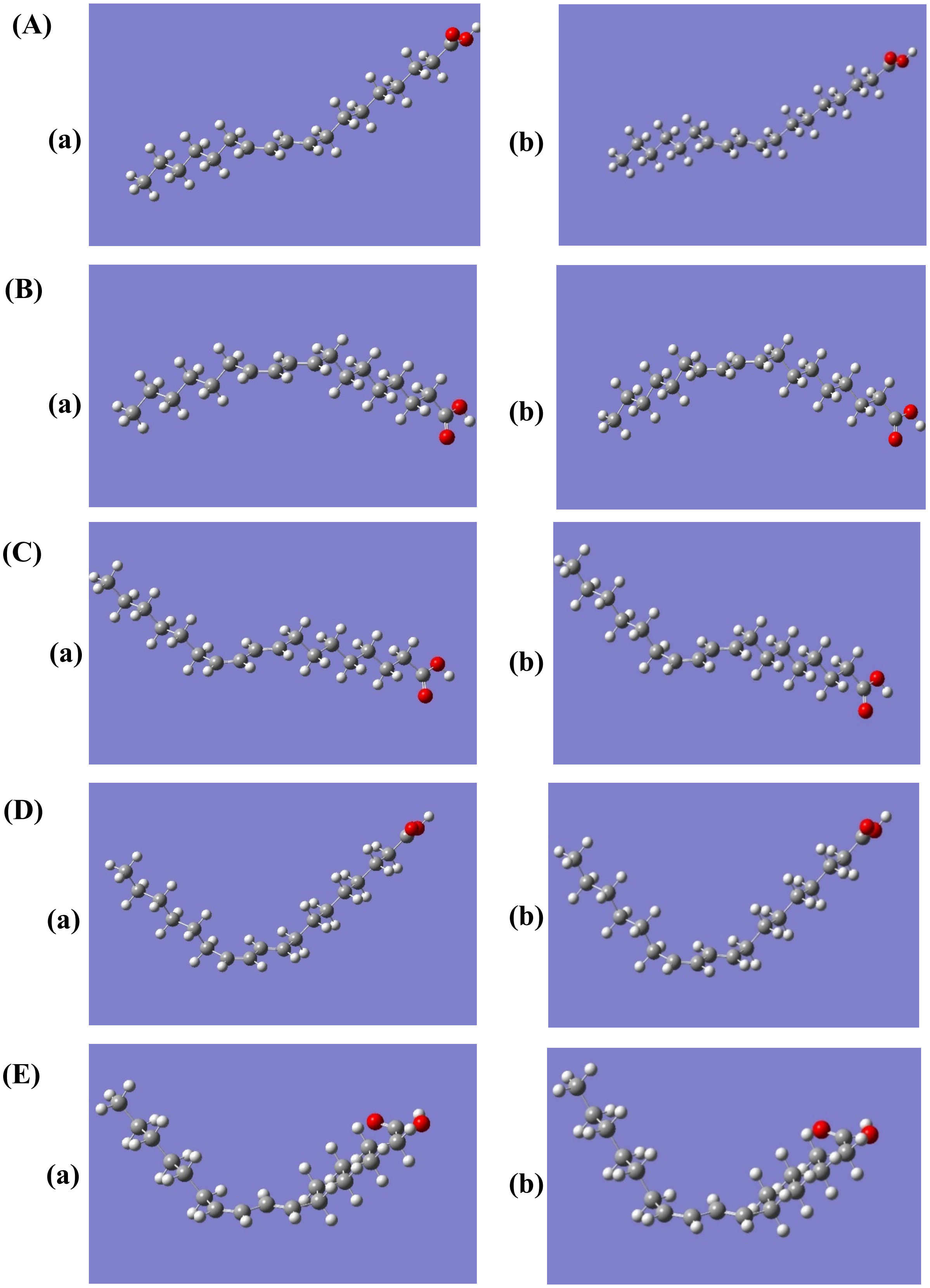

Figure S10 Structures of various conformers (A), (B), (C), (D), and (E) of the (9*E*,11*Z*)-CLA with energy minimization in the gas-phase at the B3LYP/6-31+G(d) (**a**) and APFD/6-31+G(d) (**b**) level. ΔG values (kcal.mol^{-1}) and % populations of conformers (A), (B), (C), (D), and (E) are shown in Table 1.

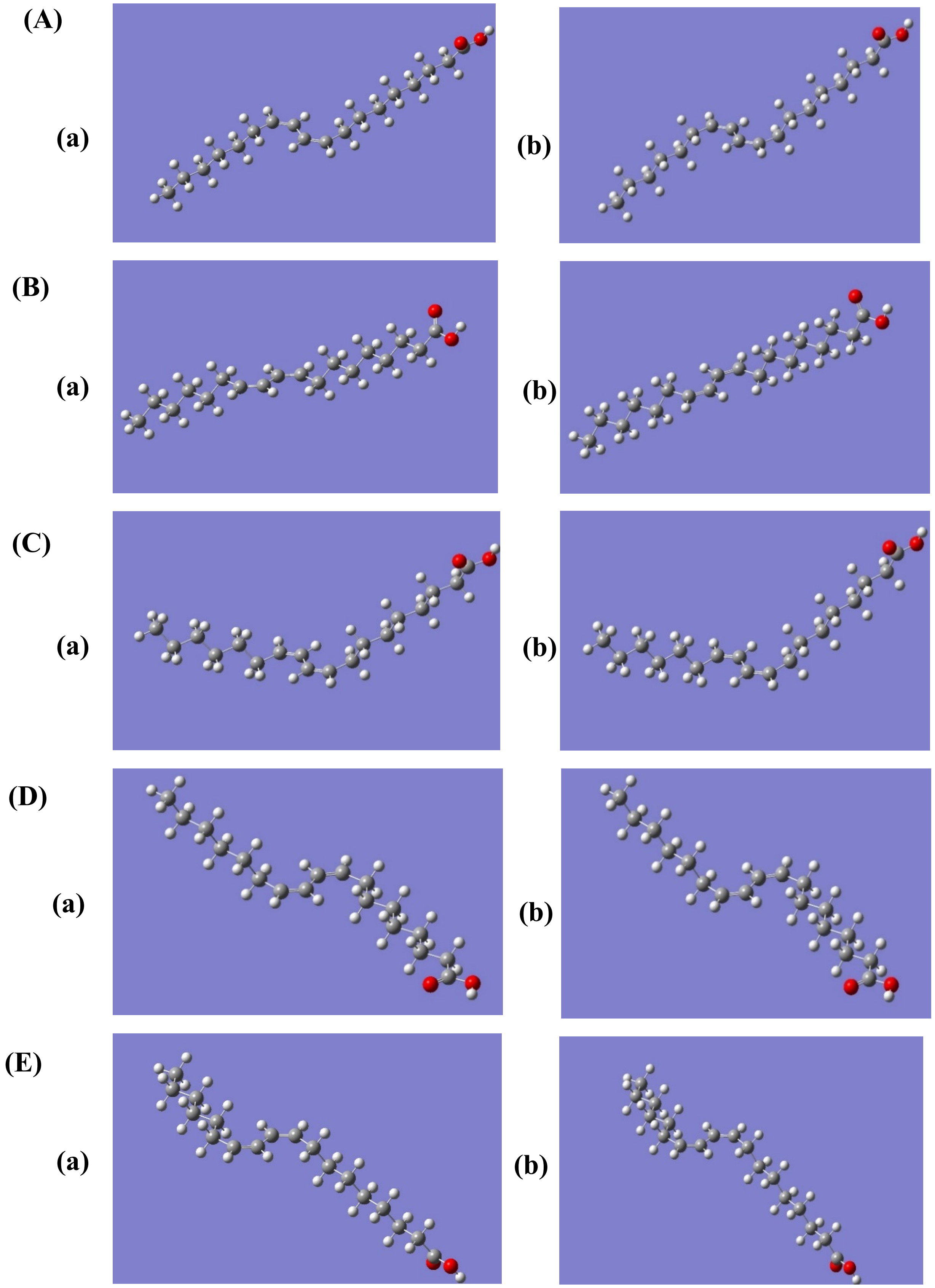

Figure 11. Structures of various conformers (A), (B), (C), (D), and (E) of the (9Z,11Z)-CLA with energy minimization in the gas-phase at the B3LYP/6-31+G(d) (**a**) and APFD/6-31+G(d) (**b**) level. ΔG values (kcal.mol^{-1}) and % populations of conformers (A), (B), (C), (D), and (E) are shown in Table 1.

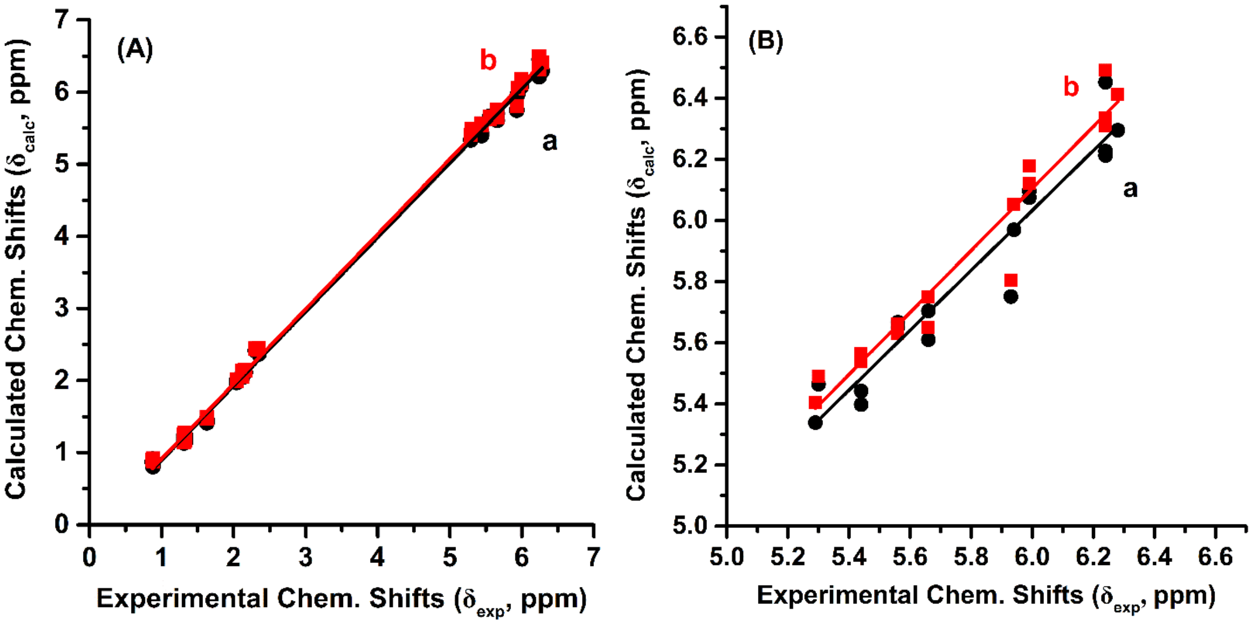

Figure 12. (A) Graphical presentation of calculated chemical shifts, weighting by the respective Boltzmann factor of the various conformers of Table 1 (at the GIAO/WP04/6-311+G(2d,p) (CPCM, $CHCl_3$) level of theory *vs.* experimental values of the ^{1}H NMR chemical shifts of the four 9,11-conjugated linoleic acid geometrical isomers with optimization of the structures at the B3LYP/6-31+G(d) (**a**), and APFD/6-31+G(d) (**b**) level of theory. (B) Olefinic region.

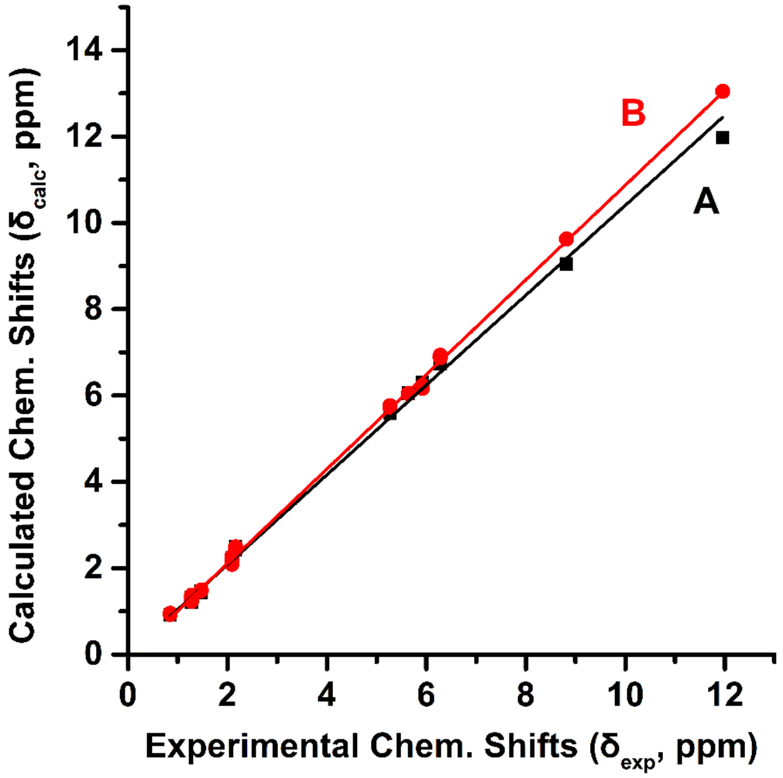

Figure 13. Graphical presentation of calculated (at the GIAO/B3LYP/6-311+G(2d,p) (CPCM) level of theory) vs. experimental values of the ^{1}H NMR chemical shifts of (9*Z*,11*E*)-Conjugated linoleic acid in DMSO-d_6 and acetonitrile-d_3, with optimization of the structures at the: (A) B3LYP/6-31+G(d) and (B) APFD/6-31+G(d) level of theory. Statistical analysis of the data has as follows: A (R^2: 0.997, slope: 1.041, intercept: -0.002); B (R^2: 0.999,

slope: 1.096, intercept: -0.087).

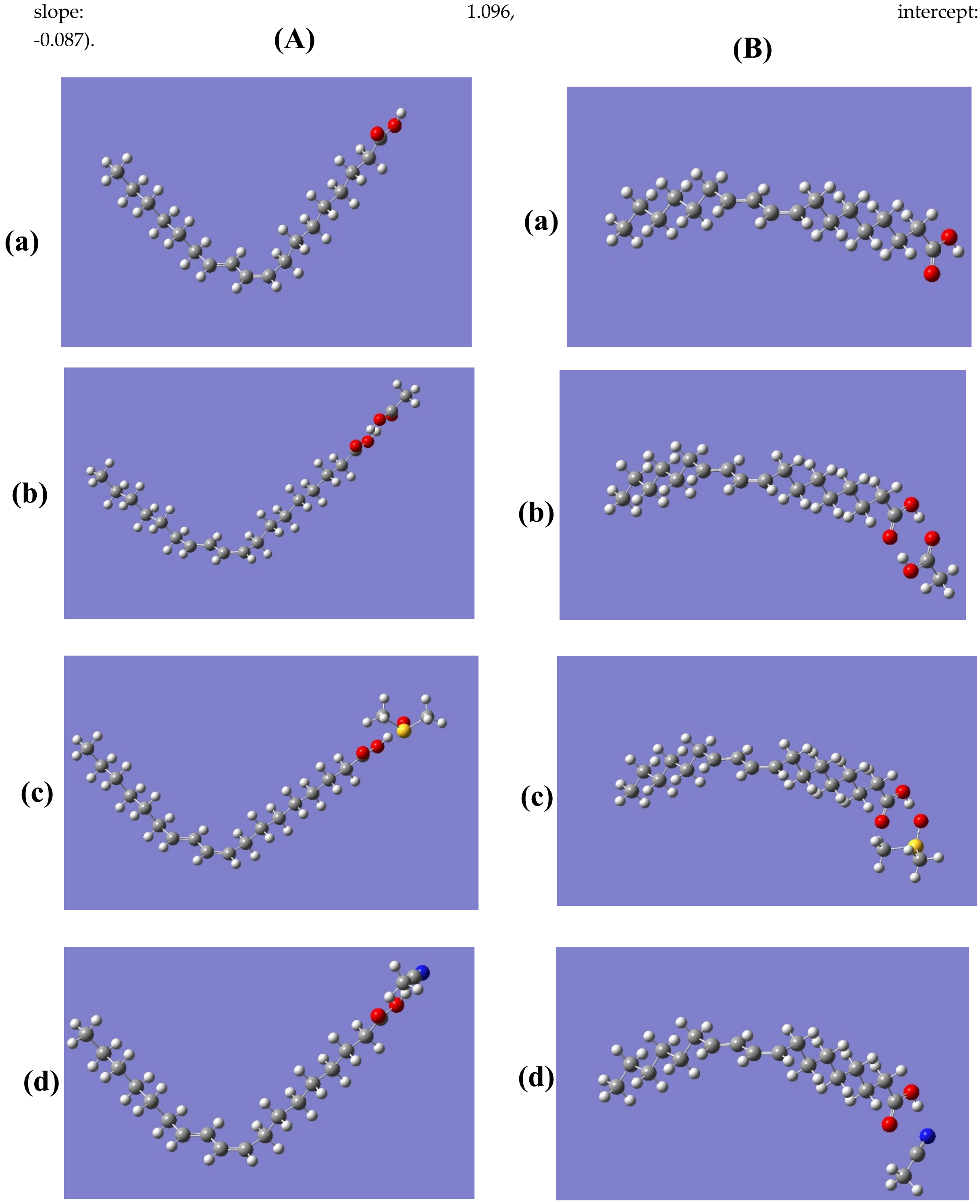

Figure 14. Effects of solvation on the structures of the low energy conformer D (Fig. S7, Table 1) of the (9*Z*,11*E*)-CLA in the gas-phase (**a**), and with a discrete molecule of CH_3COOH (**b**), DMSO (**c**) and CH_3CN (**d**), with energy minimization at the B3LYP/6-31+G(d) (**A**) and APFD/6-31+G(d) (**B**) level.

Table 1. Calculated,δ_{calc}, and experimental, δ_{exp}, ^{1}H-NMR chemical shifts of (*Z*)-1,3- pentadiene, (*E*)-1,3-pentadiene, (*E,Z*)-2,4-hexadiene using the CPCM model in $CHCl_3$ and (*E,E*)-2,4-nonadiene, (*E,Z*)-2,4-nonadiene, (*Z,E*)-2,4-nonadiene, (*Z,Z*)-2,4-nonadiene using the CPCM model in CH_3CN.

Compound	Solvent	Group	δ_{exp}	δ_{calc}									
				B3LYP/6-31+G(d)	B3LYP/6-311++G(d,p)	APFD/6-31+G(d)	APFD/6-311++G(d,p)	PBE0/6-31+G(d)	PBE0/6-311++G(d,p)	M06-2X/6-31+G(d)	M06-2X/6-311++G(d,p)	ωB97XD/6-31+G(d)	ωB97XD/6-311++G(d,p)
**(*Z*)-1,3-pentadiene**	**$CDCl_3$ [38]**	**C(1)-CH1a**	5.08	5.28	5.30	5.30	5.33	5.28	5.29	5.31	5.32	5.27	5.30
		**C(1)-CH1b**	5.16	5.42	5.44	5.46	5.48	5.43	5.45	5.48	5.49	5.43	5.46
		**C(2)-CH**	6.66	7.19	7.21	7.26	7.29	7.21	7.24	7.26	7.27	7.19	7.22
		**C(3)-CH**	6.01	6.36	6.37	6.39	6.42	6.35	6.37	6.39	6.39	6.34	6.36
		**C(4)-CH**	5.51	5.87	5.90	5.91	5.94	5.87	5.89	5.90	5.91	5.86	5.88
		**C(5)-CH_3**	1.75	1.92	1.92	1.95	1.97	1.93	1.94	1.93	1.92	1.87	1.92
**(*E*)-1,3-pentadiene**	**$CDCl_3$ [38]**	**C(1)-CH1a**	4.93	5.08	5.09	5.09	5.15	5.07	5.08	5.10	5.11	5.07	5.09
		**C(1)-CH1b**	5.06	5.29	5.30	5.32	5.25	5.29	5.31	5.34	5.35	5.30	5.32
		**C(2)-CH**	6.29	6.82	6.83	6.86	6.61	6.83	6.84	6.85	6.86	6.81	6.83
		**C(3)-CH**	6.06	6.53	6.55	6.58	6.37	6.54	6.55	6.58	6.58	6.53	6.54
		**C(4)-CH**	5.70	6.19	6.21	6.23	6.10	6.20	6.22	6.24	6.25	6.19	6.22
		**C(5)-CH_3**	1.74	1.88	1.89	1.91	1.85	1.89	1.90	1.89	1.88	1.87	1.88
**(*E,Z*)-2,4- -hexadiene**	**$CDCl_3$ [38]**	**C(1)-CH_3**	1.77	1.90	1.90	1.92	1.94	1.91	1.92	1.90	1.90	1.88	1.89
		**C(2)-CH**	5.65	6.08	6.10	6.12	6.14	6.09	6.10	6.14	6.15	6.08	6.10
		**C(3)-CH**	6.34	6.87	6.89	6.94	6.98	6.89	6.72	6.95	6.95	6.88	6.91
		**C(4)-CH**	5.96	6.29	6.30	6.33	6.35	6.29	6.30	6.32	6.33	6.27	6.29

		C(5)-CH	5.34	5.62	5.65	5.65	5.68	5.62	5.64	5.65	5.66	5.61	5.64
		C(6)-CH_3	1.72	1.88	1.88	1.91	1.94	1.89	1.91	1.89	1.88	1.86	1.88
**(*E,E*)- 2,4-nonadiene**	**CD_3CN [39]**	C(1)-CH_3	1.70	1.85	1.85	1.85	1.59	1.86	1.87	1.85	1.85	1.84	1.85
		C(2)-CH	5.57	6.02	6.04	6.01	5.84	6.02	6.04	6.06	6.07	5.95	5.98
		C(3)-CH	6.00	6.48	6.49	6.48	6.26	6.48	6.50	6.52	6.52	6.37	6.39
		C(4)-CH	6.00	6.50	6.51	6.46	6.29	6.52	6.53	6.52	6.51	6.44	6.46
		C(5)-CH	5.56	6.00	6.01	5.98	5.83	6.00	6.02	6.04	6.05	5.96	5.98
		C(6)-CH_2	2.04	2.05	2.06	2.06	1.80	2.07	2.08	2.08	2.07	2.09	2.11
		C(7)-CH_2	1.35	1.28	1.24	1.22	1.03	1.23	1.24	1.26	1.26	1.26	1.27
		C(8)-CH_2	1.31	1.36	1.36	1.36	1.13	1.35	1.36	1.37	1.37	1.35	1.36
		C(9)-CH_3	0.89	0.96	0.96	0.95	0.74	0.97	0.97	0.98	0.97	0.97	0.98
**(*E,Z*)-2,4-nonadiene**	**CD_3CN [39]**	C(1)-CH_3	1.75	1.90	1.91	1.53	1.95	1.91	1.92	1.91	1.90	1.89	1.88
		C(2)-CH	5.68	6.10	6.12	5.84	6.17	6.11	6.12	6.17	6.17	6.11	6.21
		C(3)-CH	6.37	6.86	6.89	6.54	6.98	6.88	6.91	6.95	6.95	6.89	6.91
		C(4)-CH	5.94	6.28	6.30	5.97	6.34	6.28	6.30	6.30	6.30	6.27	6.40
		C(5)-CH	5.29	5.62	5.65	5.35	5.74	5.61	5.64	5.69	5.80	5.62	5.78
		C(6)-CH_2	2.16	2.25	2.26	1.89	2.31	2.22	2.29	2.28	2.26	2.23	2.21
		C(7)-CH_2	1.34	1.23	1.23	0.91	1.25	1.22	1.23	1.25	1.24	1.21	1.20
		C(8)-CH_2	1.31	1.42	1.42	1.09	1.47	1.42	1.43	1.44	1.42	1.41	1.38
		C(9)-CH_3	0.90	0.98	0.97	0.64	1.01	0.98	0.98	0.99	0.99	0.97	0.99

**(*Z,E*)- 2,4-nonadiene**	**CD_3CN [39]**	**C(1)-CH_3**	1.71	1.90	1.90	1.92	1.96	1.91	1.93	1.91	1.89	1.88	1.90
		**C(2)-CH**	5.36	5.63	5.65	5.65	5.70	5.62	5.64	5.65	5.67	5.65	5.68
		**C(3)-CH**	5.96	6.36	6.38	6.39	6.43	6.36	6.38	6.39	6.40	6.30	6.32
		**C(4)-CH**	6.36	6.89	6.91	6.95	7.00	6.91	6.94	6.97	6.98	6.86	6.89
		**C(5)-CH**	5.68	6.11	6.13	6.14	6.18	6.12	6.13	6.17	6.18	6.11	6.12
		**C(6)-CH_2**	2.11	2.15	2.16	2.34	2.20	2.16	2.18	2.18	2.17	2.12	2.13
		**C(7)-CH_2**	1.38	1.26	1.27	1.27	1.30	1.26	1.27	1.30	1.29	1.22	1.23
		**C(8)-CH_2**	1.33	1.37	1.38	1.39	1.42	1.38	1.39	1.40	1.39	1.36	1.37
		**C(9)-CH_3**	0.90	0.98	0.97	0.99	1.01	0.98	0.99	0.99	0.99	0.97	0.98
**(*Z,Z*)- 2,4-nonadiene**	**CD_3CN [39]**	**C(1)-CH_3**	1.72	1.90	1.90	1.92	1.95	1.91	1.92	1.57	1.90	1.90	1.89
		**C(2)-CH**	5.52	5.84	5.87	5.87	5.91	5.83	5.86	5.62	5.92	5.84	5.87
		**C(3)-CH**	6.31	6.70	6.72	6.74	6.79	6.72	6.75	6.45	6.74	6.68	6.71
		**C(4)-CH**	6.27	6.69	6.72	6.73	6.77	6.70	6.73	6.42	6.75	6.67	6.70
		**C(5)-CH**	5.47	5.84	5.87	5.89	5.94	5.84	5.87	5.63	6.00	5.87	5.89
		**C(6)-CH_2**	2.17	2.27	2.28	2.30	2.33	2.29	2.31	1.95	2.26	2.25	2.27
		**C(7)-CH_2**	1.35	1.23	1.22	1.21	1.24	1.22	1.23	0.97	1.25	1.20	1.72
		**C(8)-CH_2**	1.33	1.40	1.41	1.42	1.45	1.41	1.42	1.12	1.40	1.14	1.40
		**C(9)-CH_3**	0.90	0.97	0.96	0.98	1.01	0.97	0.98	0.69	0.98	0.97	1.01

Table 2. Linear regression correlation coefficient (R^2), mean square error, intercept and slope of: (A) calculated, δ_{calc}, *vs.* experimental, δ_{exp}, ^1H chemical shifts of the model compounds of Figure 2 determined from various optimized geometries and (B) δ_{calc}, *vs.*δ_{exp}, of the olefinic ^1H-NMR chemical shifts of 1(A).

Method	(R^2)	Mean square error	Intercept	Slope	(R^2)	Mean square error	Intercept	Slope
	(A)				(B)			
B3LYP/6-31+G(d)	0.999	0.007	-0.058	1.078	0.987	0.004	-0.688	1.187
B3LYP/6-311++G(d,p)	0.999	0.007	-0.062	1.083	0.987	0.004	-0.675	1.188
APFD/6-31+G(d)	0.996	0.025	-0.129	1.089	0.941	0.018	-0.694	1.186
APFD/6-311++G(d,p)	0.997	0.018	-0.085	1.086	0.968	0.009	-0.632	1.180
PBE0/6-31+G(d)	0.999	0.008	-0.053	1.079	0.987	0.004	-0.775	1.203
PBE0/6-311++G(d,p)	0.999	0.008	-0.042	1.079	0.984	0.005	-0.672	1.188
M06-2X/6-31+G(d)	0.996	0.022	-0.131	1.091	0.938	0.019	-0.593	1.171
M06-2X/6-311++G(d,p)	0.999	0.008	-0.067	1.091	0.979	0.006	-0.603	1.183
ωB97XD/6-31+G(d)	0.999	0.008	-0.083	1.081	0.986	0.004	-0.635	1.176
ωB97XD/6-311++G(d,p)	0.998	0.009	-0.035	1.078	0.981	0.005	-0.559	1.168

Table S3. Calculated, δ_{calc}, and experimental, δ_{exp}, ^{13}C NMR chemical shifts of (*Z*)-1,3-pentadiene, (*E*)-1,3-pentadiene, (*E,Z*)-2,4-hexadiene using the CPCM model in $CHCl_3$ and (*E,E*)-2,4-nonadiene, (*E,Z*)-2,4-nonadiene, (*Z,E*)-2,4-nonadiene, (*Z,Z*)-2,4-nonadiene using the CPCM model in CH_3CN.

**Compound**	**Solvent**	**Group**	δ_{exp}	δ_{calc}									
				**B3LYP/6-31+G(d)**	**B3LYP/6-311++G(d,p)**	**APFD/6-31+G(d)**	**APFD/6-311++G(d,p)**	**PBE0/6-31+G(d)**	**PBE0/6-311++G(d,p)**	**M062X/6-31+G(d)**	**M062X/6-311++G(d,p)**	**ωB97XD/6-31+G(d)**	**ωB97XD/6-311++G(d,p)**
**(Z)-1,3 pentadiene**	**$CDCl_3$ [38]**	**C(1)**	116.60	120.98	120.72	121.13	121.12	120.85	120.61	121.01	120.81	120.73	120.46
		**C(2)**	132.14	140.82	140.58	140.79	140.81	140.44	140.12	140.93	140.78	140.57	140.32
		**C(3)**	130.27	136.86	136.59	136.97	136.97	136.54	136.28	137.12	136.90	136.63	136.38
		**C(4)**	126.81	138.12	137.89	138.01	138.05	137.75	137.54	137.52	138.33	137.39	137.13
		**C(5)**	13.34	15.93	15.93	16.06	16.35	15.83	15.87	161.03	16.11	16.03	16.02
**(E)-1,3 pentadiene**	**$CDCl_3$ [38]**	**C(1)**	114.38	118.60	118.33	118.73	118.55	118.38	118.14	118.41	118.18	118.23	117.98
		**C(2)**	137.36	146.83	146.63	146.93	145.67	146.63	146.46	147.08	146.96	146.73	146.51
		**C(3)**	132.57	140.03	139.78	140.24	139.27	139.79	139.58	140.43	140.26	139.80	139.57
		**C(4)**	129.76	141.63	141.40	141.64	140.64	141.44	141.23	141.25	141.03	141.07	140.81
		**C(5)**	17.99	21.17	21.19	21.36	21.52	21.12	21.17	21.39	21.37	21.32	21.33
**(E,Z)-2,4 hexadiene**	**$CDCl_3$ [38]**	**C(1)**	18.27	21.32	21.35	21.54	21.87	21.30	21.27	21.59	21.59	21.48	21.51
		**C(2)**	128.81	139.06	138.77	138.97	138.93	138.81	138.53	139.00	138.84	138.70	138.37

		**C(3)**	126.93	133.52	133.27	133.56	133.59	133.16	132.92	133.63	133.44	133.11	132.81
		**C(4)**	129.58	136.39	136.12	136.43	136.44	136.07	135.82	136.51	136.35	136.15	135.85
		**C(5)**	123.60	133.19	132.96	132.06	133.10	132.74	132.53	132.67	132.47	132.56	132.32
		**C(6)**	13.22	15.72	15.72	15.84	16.15	15.63	15.67	15.45	15.82	15.78	15.83
**(E,E)-2,4 Nonadiene**	**CD_3CN [39]**	**C(1)**	18.180	21.13	21.20	21.14	20.20	21.14	21.20	21.31	21.37	21.40	21.41
		**C(2)**	127.63	137.01	136.78	136.37	134.56	136.73	136.49	136.63	136.50	136.74	136.47
		**C(3)**	132.77	139.49	139.30	139.17	137.92	139.45	139.14	139.69	139.55	138.66	138.46
		**C(4)**	131.38	137.88	137.71	137.25	137.27	137.68	137.49	137.88	137.75	137.17	136.99
		**C(5)**	133.04	142.84	142.63	142.43	140.78	142.72	142.47	142.66	142.54	142.40	142.14
		**C(6)**	32.92	40.49	40.56	40.53	39.49	40.44	40.49	40.85	40.88	40.75	40.75
		**C(7)**	32.41	39.24	39.29	39.75	37.86	39.30	39.32	39.83	39.94	39.43	39.41
		**C(8)**	22.99	29.58	29.58	29.64	28.38	29.49	29.48	29.86	29.86	29.59	29.53
		**C(9)**	14.25	16.67	16.66	16.71	15.57	16.82	16.82	16.90	16.88	17.05	17.00
**(E,Z)-2,4 Nonadiene**	**CD_3CN [39]**	**C(1)**	18.42	21.37	21.45	19.42	17.95	21.39	21.45	21.59	21.67	21.61	15.95
		**C(2)**	130.04	139.59	139.32	136.66	139.45	139.36	139.08	139.55	139.49	139.19	134.44
		**C(3)**	128.09	133.64	133.44	131.15	133.80	133.38	133.16	133.83	133.60	133.36	127.54
		**C(4)**	129.62	135.08	134.89	132.80	135.17	134.72	134.51	135.09	135.39	134.93	129.61
		**C(5)**	130.68	139.67	139.49	136.94	139.72	139.45	139.22	139.29	138.84	139.13	133.80
		**C(6)**	28.02	34.43	34.48	32.52	34.89	34.33	34.35	34.76	34.78	34.57	27.37
		**C(7)**	23.06[a]	38.89	38.91	36.55	39.83	38.96	38.97	39.59	39.73	39.27	31.65
		**C(8)**	32.74[a]	29.74	29.73	27.52	30.19	29.64	29.63	29.2	29.88	29.85	22.67

		**C(9)**	14.29	16.58	16.58	14.44	17.18	16.73	16.73	16.85	16.96	16.85	11.20
**(Z,E)-2,4 Nonadiene**	**CD_3CN [39]**	**C(1)**	13.44	15.75	15.80	15.74	16.16	15.70	15.74	15.91	15.94	15.80	15.81
		**C(2)**	124.75	134.51	134.34	134.15	134.33	134.10	133.90	133.88	133.74	133.51	133.27
		**C(3)**	130.58	136.60	136.37	136.56	136.69	136.3	136.07	136.71	136.57	136.22	135.93
		**C(4)**	126.42	132.13	131.95	132.00	132.18	131.72	131.51	131.96	131.91	131.79	131.57
		**C(5)**	135.55	145.53	145.30	145.49	145.58	145.48	145.20	145.72	145.65	144.92	144.57
		**C(6)**	33.19	40.86	40.96	41.02	41.47	40.84	40.90	41.23	41.28	40.85	40.86
		**C(7)**	32.39	39.13	39.16	39.73	40.13	39.20	39.21	39.67	39.78	39.99	40.01
		**C(8)**	23.03	29.51	29.50	29.60	29.96	29.43	29.42	29.76	29.75	29.49	29.43
		**C(9)**	14.26	16.75	16.74	17.03	17.40	16.89	16.89	17.02	17.05	16.88	16.83
**(Z,Z)-2,4 Nonadiene**	**CD_3CN [39]**	**C(1)**	13.31	15.43	15.49	15.46	15.89	15.40	15.44	13.70	15.62	15.50	15.53
		**C(2)**	126.87	136.30	136.10	135.93	136.08	135.92	135.69	133.60	135.92	135.76	135.50
		**C(3)**	125.54	130.38	130.13	130.19	130.31	129.98	129.72	128.21	129.97	129.91	129.64
		**C(4)**	124.30	128.70	128.55	128.51	128.72	128.17	127.96	126.39	129.02	128.34	128.08
		**C(5)**	132.87	142.13	141.90	141.78	141.85	142.93	141.66	139.77	141.17	141.50	141.24
		**C(6)**	27.84	33.99	33.99	33.92	34.33	33.87	33.86	32.34	34.41	33.95	33.92
		**C(7)**	32.61	38.78	38.83	39.26	396.58	38.86	38.88	36.71	39.60	39.20	39.19
		**C(8)**	23.07	29.59	29.58	29.66	300.20	29.50	29.49	27.59	29.82	29.67	29.62
		**C(9)**	14.27	16.62	16.58	16.79	171.69	16.76	16.75	14.75	16.88	16.84	16.80

[a] The assignment of the experimental chemical shifts should be reversed

Table S4. Linear regression correlation coefficient, mean square error, intercept and slope of calculated vs. experimental ^{13}C chemical shifts of the models compounds determined from various minimized geometries.

Method	Correlation coefficient R^2	Mean square error	Intercept	Slope
B3LYP/6-31+G(d)	0.997	7.876	3.799	1.032
B3LYP/6-311++G(d,p)	0.997	7.926	3.873	1.030
APFD/6-31+G(d)	0.997	8.606	3.538	1.030
APFD/6-311++G(d,p)	0.997	8.427	3.932	1.028
PBE0/6-31+G(d)	0.997	8.053	3.839	1.030
PBE0/6-311++G(d,p)	0.997	8.010	3.917	1.027
M06-2X/6-31+G(d)	0.997	8.968	3.661	1.030
M06-2X/6-311++G(d,p)	0.997	8.197	4.237	1.027
*ω*B97XD/6-31+G(d)	0.997	7.920	4.104	1.026
*ω*B97XD/6-311++G(d,p)	0.996	12.742	2.674	1.030

Table S5. Linear regression correlation coefficient, mean square error, intercept and slope of calculated *vs.* experimental olefinic ^{13}C chemical shifts of (*Z*)-1,3-pentadiene, (*E*)-1,3-pentadiene, and (*E,Z*)-2.4-hexadiene, (*E,E*)-2,4-nonediene, (*Z,Z*)-2,4-nonediene, (*E,Z*)- 2,4-nonediene, and (*Z,E*)- 2,4-nonediene (Fig. 1) determined from various minimized geometries.

Method	Correlation coefficient (R^2)	Mean square error	Intercept	Slope
B3LYP/6-31+G(d)	0.895	4.144	-18.642	1.206
B3LYP/6-311++G(d,p)	0.896	4.125	-18.990	1.207
APFD/6-31+G(d)	0.873	5.015	-17.138	1.190
APFD/6-311++G(d,p)	0.892	3.961	-13.154	1.160
PBE0/6-31+G(d	0.895	4.252	-20.778	1.220
PBE0/6-311++G(d,p)	0.896	4.175	-20.073	1.213
M06-2X/6-31+G(d)	0.894	4.446	-23.743	1.242
M06-2X/6-311++G(d,p)	0.911	3.568	-20.841	1.221
*ω*B97XD/6-31+G(d)	0.903	3.812	-19.158	1.207
*ω*B97XD/6-311++G(d,p)	0.805	8.135	-16.171	1.176

Table S6. Calculated chemical shifts, δ_{calc} (φ=31.0°) and δ_{calc} (φ=180.0°), of the two low energy conformers of (*E*)-1,3–pentadiene due to variation of the $C_1C_2C_3C_4$ torsion angle (Fig. 4), and their chemical shifts, δ_{calc} (w), weighting by the respective Boltzmann factor (in parenthesis are their populations).

**Group**	**δ_{calc} (φ=31.0°) ppm (0.32%)**	**δ_{calc} (φ=180.0°) ppm (99.68%)**	**δ_{calc} (w), ppm**
**C(1)-CH1a**	5.09	5.08	5.08
**C(1)-CH1b**	5.64	5.29	5.29
**C(2)-CH**	6.57	6.82	6.82
**C(3)-CH**	6.28	6.53	6.53
**C(4)-CH**	6.50	6.20	6.19
**C(5)-CH_3**	1.87	1.88	1.88

Table S7. Calculated chemical shifts, δ_{calc} (φ) of the low energy conformers due to variation of three torsion angles of *(E,E)*-2,4-nonediene (Figs. 5, 6, and 7), and their chemical shifts, δ_{calc} (w), weighting by the respective Boltzmann factor (in parenthesis are their populations).

	**Torsion angle $C_2C_3C_4C_5$**			**Torsion angle $C_4C_5C_6C_7$**			**Torsion angle $C_6C_7C_8C_9$**		
**Group**	**δ_{calc} (φ=31.2°) ppm (0.32%)**	**δ_{calc} (φ=180.0°) ppm (99.68%)**	**δ_{calc} (w), ppm**	**δ_{calc} (φ=0.1°) ppm (11.55%)**	**δ_{calc} (φ=120.0°) ppm (85.45%)**	**δ_{calc} (w), ppm**	**δ_{calc} (φ=64.8°) ppm (18.74%)**	**δ_{calc} (φ=180.0°) ppm (81.26%)**	**δ_{calc} (w), ppm**
**C(1)-CH_3**	1.84	1.85	1.85	1.85	1.85	1.85	1.85	1.85	1.85
**C(2)-CH**	6.32	6.02	6.02	6.00	6.02	6.01	6.00	6.02	6.01
**C(3)-CH**	6.24	6.48	6.47	6.41	6.48	6.47	6.15	6.48	6.46
**C(4)-CH**	6.08	6.50	6.50	6.28	6.50	6.47	6.34	6.50	6.47
**C(5)-CH**	6.25	6.00	6.00	6.24	6.00	6.02	5.94	6.00	5.99
**C(6)-CH_2**	2.13	2.05	2.05	2.25	2.05	2.08	2.09	2.05	2.06
**C(7)-CH_2**	1.22	1.28	1.28	1.40	1.28	1.30	1.36	1.28	1.30
**C(8)-CH_2**	1.35	1.36	1.36	1.33	1.36	1.36	1.49	1.36	1.39
**C(9)-CH_3**	0.96	0.96	0.96	1.00	0.96	0.96	0.93	0.96	0.95

Table S8. Calculated (δ_{calc}, ppm) and experimental (δ_{exp}, ppm) ^{1}H-NMR chemical shifts of the 9,11-conjugated linoleic acid (CLA) geometric isomers with geometry optimization at the B3LYP/6-31+G(d) and APFD/6-31+G(d) level.

**Compound**	**Atom**	**$\delta_{exp.}$ (ppm)**	**B3LYP/6-31+G(d) δ_{calc}(ppm)**	**APFD/6-31+G(d) δ_{calc}(ppm)**
(9*Z*,11*E*)-CLA	H11	6.28	6.30	6.41
	H10	5.94	5.97	6.05
	H9	5.29	5.34	5.40
	H12	5.66	5.61	5.65
	H2	2.35	2.37	2.44
	H8	2.12	2.10	2.13
	H13	2.12	2.04	2.05
	H3	1.63	1.46	1.49
	H4	1.33	1.19	1.19
	H5	1.33	1.21	1.24
	H6	1.33	1.23	1.26
	H7	1.33	1.18	1.18
	H14	1.33	1.24	1.26
	H15	1.33	1.23	1.24
	H16	1.33	1.15	1.17
	H17	1.33	1.20	1.23
	H18	0.88	0.85	0.88
(9*Z*,11*Z*)-CLA	H11	6.24	6.23	6.31
	H10	6.24	6.21	6.33
	H9	5.44	5.40	5.54
	H12	5.44	5.44	5.57
	H2	2.35	2.37	2.45
	H8	2.16	2.11	2.14
	H13	2.16	2.11	2.15
	H3	1.63	1.42	1.49
	H4	1.32	1.16	1.17
	H5	1.32	1.21	1.22
	H6	1.32	1.24	1.26
	H7	1.32	1.19	1.21
	H14	1.32	1.18	1.20
	H15	1.32	1.21	1.24
	H16	1.32	1.15	1.17
	H17	1.32	1.27	1.27
	H18	0.88	0.81	0.89
(9*E*,11*E*)-CLA	H11	5.99	6.10	6.12
	H10	5.99	6.07	6.18
	H9	5.56	5.67	5.66
	H12	5.56	5.65	5.63
	H2	2.34	2.41	2.43
	H8	2.04	1.99	1.99
	H13	2.04	1.97	2.02
	H3	1.63	1.46	1.47
	H4	1.31	1.13	1.15
	H5	1.31	1.20	1.22
	H6	1.31	1.22	1.25
	H7	1.31	1.25	1.25
	H14	1.31	1.22	1.24
	H15	1.31	1.17	1.24
	H16	1.31	1.14	1.14

	H17	1.31	1.22	1.19
	H18	0.87	0.87	0.90
(9*E*,11*Z*)-CLA	H11	5.93	5.75	5.80
	H10	6.24	6.45	6.49
	H9	5.66	5.70	5.75
	H12	5.30	5.46	5.49
	H2	2.3	2.42	2.44
	H18	0.88	0.91	0.92

Table S9. Solvent effects on the ^1H NMR chemical shifts of (9*Z*,11*E*)-CLA in $CDCl_3$, CD_3CN and DMSO-d_6 at 298K.

Group	$CDCl_3$	CD_3CN	DMSO-d_6
-COOH	10.50	8.82	11.96
H11	6.28	6.34	6.28
H10	5.94	5.94	5.92
H12	5.66	5.66	5.64
H9	5.29	5.29	5.27
H2	2.35	2.25	2.17
H8,13	2.12	2.12	2.09
H3	1.63	1.55	1.47
H4-7,14-17	1.33	1.33	1.28
H18	0.88	0.88	0.85

Table S10. Statistical analysis of the data of Figure S12.

	R^2	Slope	Intercept
(A) 1(a)	0.999	1.030	-0.135
(A) 1(b)	0.999	1.040	-0.125
(B) 2(a)	0.927	0.980	0.154
(B) 2(b)	0.943	1.014	0.021
